# Transcriptomics in *Erigeron canadensis* reveals rapid photosynthetic and hormonal responses to auxin herbicide application

**DOI:** 10.1093/jxb/eraa124

**Published:** 2020-03-12

**Authors:** Cara L McCauley, Scott A M McAdam, Ketaki Bhide, Jyothi Thimmapuram, Jo Ann Banks, Bryan G Young

**Affiliations:** 1 Department of Botany and Plant Pathology, Purdue University, West Lafayette, IN, USA; 2 Bioinformatics Core, Purdue University, West Lafayette, IN, USA; 3 University of Warwick, UK

**Keywords:** Abscisic acid, auxin, dicamba, 2,4-D, *Erigeron canadensis*, ethylene, halauxifen-methyl, herbicide

## Abstract

The perception pathway for endogenous auxin has been well described, yet the mode of action of synthetic auxin herbicides, used for >70 years, remains uncharacterized. We utilized transcriptomics and targeted physiological studies to investigate the unknown rapid response to synthetic auxin herbicides in the globally problematic weed species *Erigeron canadensis*. Synthetic auxin herbicide application consistently and rapidly down-regulated the photosynthetic machinery. At the same time, there was considerable perturbation to the expression of many genes related to phytohormone metabolism and perception. In particular, auxin herbicide application enhanced the expression of the key abscisic acid biosynthetic gene, *9-cis-epoxycarotenoid deoxygenase* (*NCED*). The increase in *NCED* expression following auxin herbicide application led to a rapid biosynthesis of abscisic acid (ABA). This increase in ABA levels was independent of a loss of cell turgor or an increase in ethylene levels, both proposed triggers for rapid ABA biosynthesis. The levels of ABA in the leaf after auxin herbicide application continued to increase as plants approached death, up to >3-fold higher than in the leaves of plants that were drought stressed. We propose a new model in which synthetic auxin herbicides trigger plant death by the whole-scale, rapid, down-regulation of photosynthetic processes and an increase in ABA levels through up-regulation of *NCED* expression, independent of ethylene levels or a loss of cell turgor.

## Teaser

Auxin herbicides trigger a rapid down-regulation of photosynthetic-related gene expression and an up-regulation of abscisic acid biosynthesis independent of other plant hormones in *Erigeron*.

## Introduction

Auxin is essential for plant growth and development, and the perception and signaling pathway for this hormone is well described (Hagen and Guilfoyle, 2002; Dharmasiri *et al.*, 2005a; Kepinski and Leyser, 2005; Tan *et al.*, 2007). In contrast, synthetic auxins have been used as herbicides for >70 years, yet, unlike endogenous auxin, the precise mode of action leading to plant death by these compounds remains unknown. The signaling pathway for endogenous auxin is believed to be exploited by synthetic auxin herbicides to trigger plant death (Grossmann, 2010; Peterson *et al.*, 2016). Attempts to demystify the herbicidal mode of action of auxins have focused intensively on the auxin-binding TRANSPORT INHIBITOR RESPONSE 1 (TIR1)/AUXIN SIGNALING F-BOX (AFB) receptors in *Arabidopsis thaliana* L. (Brassicaceae). The *TIR1*/*AFB* gene family consists of six receptors: *TIR1* and five homologs of *AFB* (Dharmasiri *et al.*, 2005b; Parry *et al.*, 2009; Prigge *et al.*, 2016). Analysis of *A. thaliana* mutant lines has confirmed that these receptor genes are indeed essential for the plant perception and specificity of auxin herbicides. The *afb5* mutant is known to be resistant to chlorinated picolinic acid (picloram) and 3,6-dichloro-2-methoxybenzoic acid (dicamba), both synthetic auxins, but not to 2,4-dichlorophenoxyacetic acid (2,4-D), another synthetic auxin, or the endogenous auxin, indole-3-acetic acid (IAA) (Walsh *et al.*, 2006; Gleason *et al.*, 2011). In contrast, *tir1* mutants are resistant to 2,4-D and dicamba (Gleason *et al.*, 2011). A further indicator of auxin herbicide action in plants is the rapid increase in expression of the auxin-responsive gene *GH3* (Goda *et al.*, 2004; Kelley and Riechers, 2007). While this research has been instrumental in demonstrating the perception pathway of synthetic auxins, it has not revealed the specific physiological mechanisms leading to plant death that are activated by the perception of these exogenous compounds.

The IAA-induced transcriptome has been characterized in *A. thaliana* and serves as the foundation for investigating the plant response to synthetic auxin compounds (Hagen and Guilfoyle, 2002; Goda, 2004; Paponov *et al.*, 2008; Chapman and Estelle, 2009). Transcriptome studies in *A. thaliana* have found that a number of synthetic auxins trigger similar differential gene expression patterns (Pufky *et al.*, 2003). These studies have focused intensively on the downstream genes known to be responsible for regulating plant growth and development; however, among these auxin-induced genes are those associated with the metabolism and signaling of other plant hormones (Grossmann, 2010). These additional plant hormones, including abscisic acid (ABA) and ethylene, are assumed to play a fundamental role in the triggering of plant death following auxin herbicide application (Sterling and Hall, 1997; Grossmann, 2010).

The endogenous phytohormone ABA modulates many plant development processes including seed maturation, root growth, and senescence (Finkelstein, 2013). In addition, ABA plays an active role in restricting plant water loss via stomatal closure (Mittelheuser and Van Steveninck, 1969). Scheltrup and Grossmann (1995) first reported ABA accumulation in *Galium aparine* L. (Rubiaceae) following exposure to 7-chloro-3-methylquinoline-8-carboxylic acid (quinmerac), a synthetic auxin, and hypothesized that subsequent stomatal closure following herbicide application was due to increased ABA levels. Further work in *G. aparine* and *A*. *thaliana* found that exogenous IAA or 2,4-D application can lead to an increase in the expression of *9-cis-Epoxycarotenoid Dioxygenase* (*NCED*) (Raghavan *et al*., 2005; Kraft *et al.*, 2007). The enzyme NCED catalyzes the rate-limiting step in the ABA biosynthetic pathway (Qin and Zeevaart, 1999). The auxin-induced increase in *NCED* expression appears to occur without a change in cell turgor or volume decline, the trigger which commonly leads to increased ABA levels and stomatal closure during soil water deficit or at high vapor pressure deficit (VPD) (McAdam *et al.*, 2016).

In *G. aparine*, Kraft *et al.* (2007) observed auxin herbicide-induced increased expression of *1-AMINOCYCLOPROPANE-1-CARBOXYLATE SYNTHASE* (*ACS*), encoding the enzyme responsible for catalyzing the rate-limiting step in the ethylene biosynthetic pathway (Boller *et al.*, 1979), and observed an increase in ethylene levels. The auxin herbicide-induced *ACS* expression is proposed to be a primary target for the mode of action of synthetic auxins (Sterling and Hall, 1997), with increased ethylene levels leading to cell death. The up-regulation of genes encoding *ACS* has been described following IAA and dicamba treatment (Abel *et al.*, 1995; Gleason *et al.*, 2011; Pettinga *et al.*, 2018). The concurrent observations of increased *ACS* and *NCED* expression following auxin herbicide treatment led Kraft *et al.* (2007) to hypothesize that auxin-induced increases in ethylene levels were the trigger for the up-regulation of *NCED* expression. However, not all transcriptome evidence confirms this hypothesis, with Raghavan *et al*. (2005) finding that *NCED3* expression was up-regulated after treatment with 2,4-D, but that there was no change in the expression of *ACS* or *ACC OXIDASE* in *A. thaliana*. These conflicting reports indicate that further work is required to establish a valid model for auxin herbicide action, particularly regarding the involvement of ABA and ethylene.

Given these conflicting findings, probably arising from the high level of complexity in the network of plant hormone interactions and pathways perturbed by synthetic auxin herbicide application, global transcriptomic approaches provide a powerful tool for revealing the unknown mode of action of auxin herbicides. The primary objective of this research was to explore the mode of action of synthetic auxin herbicides through transcriptomics, with a particular emphasis placed on the interactive effects of these herbicides on other plant hormone bio synthetic and signaling pathways. Unlike previous studies focusing on the model system *A. thaliana* or *G. aparine*, this work specifically targeted one of the most problematic, broad-leaved weed species impacting temperate agriculture globally, *Erigeron canadensis* L. (Asteraceae) (Gibson *et al.*, 2006; Davis *et al.*, 2009). The recent evolution of *E. candensis* biotypes that are resistant to glyphosate and acetolactate synthase (ALS)-inhibiting herbicides across North America (Kruger *et al.*, 2009; VanGessel *et al.*, 2009; Heap, 2018) places an acute emphasis on understanding the mode of action of auxin herbicides as a control option for this species. The availability of a reference genome in *E. canadensis* (Peng *et al.*, 2014) facilitates large-scale transcriptomic studies.

## Materials and methods

### Plant material and growth conditions

*Erigeron canadensis* seeds, collected from Brookston, IN, USA (40.59°N, 86.76°W), were sown onto commercial potting medium (Sun Gro Propagation Mix, Sun Gro Horticulture). Plants were grown under controlled glasshouse conditions, with experiments conducted on 30-day-old plants. Temperature was maintained between 23 °C and 29 °C; supplemental light was provided with high-pressure sodium bulbs set to a 16 h photoperiod (1100 µmol m^−2^ s^−1^ photon flux density at pot level). Once seedlings reached the three- to five-leaf stage, individual seedlings were transplanted into 100 cm^2^ pots containing the same commercial potting medium. Plants were watered as needed and fertilized weekly (Jack’s Professional 20-20-20, JR Peters Inc.). Treatments included commercially labeled field rates of 2,4-D dimethylamine salt (Weedar^®^ 64, Nufarm Inc.), dicamba diglycolamine salt (Clarity®, BASF Corporation), and halauxifen-methyl (Arylex™ active, Dow AgroSciences LLC) at 560, 280, and 5 g of acid equivalent ha^−1^, respectively, in addition to a water-only treatment that served as the control. A non-ionic surfactant (Activator-90, Loveland Products) was added at 0.25% (v/v) to all treatments including the control. Herbicide applications were made to 6–10 cm rosettes using single-nozzle track-mounted sprayer with a single flat fan XR 8002E nozzle calibrated to deliver 140 l ha^−1^.

### Reference genome annotation

Gene prediction was performed on the assembled *E. canadensis* genome (accession GCA_000775935.1), using GeneMark (Borodovsky and Lomsadze, 2011), and generated protein sequences were annotated using blastp against the non-redundant protein database from NCBI and the *A. thaliana* protein database from TAIR10. Annotation for each gene was assigned according to the top blast hit with an E-value cut-off of 10^–3^. The *A. thaliana* annotation was utilized for the ssequent functional interpretation of differentially expressed genes.

### RNA extraction and whole-transcriptome sequencing

Plant tissue samples were collected at 1 h and 6 h after herbicide treatment (HAT) for whole-transcriptome sequencing. For each biological replicate, a single leaf was excised from separate plants and immediately flash-frozen in liquid nitrogen and stored at –80 °C until RNA extraction. Leaf tissue was pulverized in a lysis buffer with β-mercaptoethanol, and total RNA was extracted according to the RNeasy Plant Mini Kit protocol with slight modifications (Qiagen cat. no. 74904). To ensure removal of genomic DNA, total RNA was subjected to an RNA Clean & Concentrator kit (Zymo Research cat. no. R1013). Total RNA was submitted to the Purdue Genomics Core Facility (Purdue University) for poly(A)^+^ RNA selection, library construction, and sequencing. Samples were dual-barcoded and pooled prior to sequencing on an Illumina HiSeq2500 platform using paired-end technology in ‘rapid’ mode. Approximately 80 million paired-end reads were generated for each of the 32 samples. For RNA sequencing (RNA-seq), four biological replications, where a replication represents an individual plant, were included for each treatment at each time point. Raw data and transcriptome assemblies are accessible in the NCBI repository (BioProject PRJNA480695).

### Differential gene expression analysis

Sequence quality control and quality trimming was performed using FastQC (version 0.11.2) and the FASTX-Toolkit (version 0.0.14) with a minimum Phred33 quality score of 30. Processed reads of at least 50 bases in length were mapped to the annotated genome using the STAR aligner (version 2.5.2b) with default parameters. To generate read counts for each gene, HTSeq (Version 0.6.1) was used; custom Perl scripts were used to generate a read count matrix that included all samples and replicates. Differential gene expression analysis was performed with three different methods: edgeR and DESeq2, both of which were carried out using ‘R’ (Version 3.3.2); and Cufflinks (v 2.2.1). In edgeR (v 3.16.5), differential expression was calculated via an exact test for differences between the negative binomial distribution of counts for each herbicide treatment compared with the water-only treatment. Differentially expressed gene lists generated with DESeq2 (v 1.14.1) for each herbicide treatment used an estimated variance-mean test using the negative binomial distribution. In the Cufflinks analysis, differential gene expression analysis was performed based on fragments per kilobase of exon per million fragments mapped (FPKM) values and pairwise comparisons of these FPKM values. To optimize accuracy and breadth in the analysis, genes detected as significant [adjusted *P*-value (adj*P*) ≤0.05] in at least two of the three analyses for each treatment were included. Venn diagrams were generated for differentially expressed genes among herbicide treatments using Venny 2.1 (http://bioinfogp.cnb.csic.es/tools/venny/) (Supplementary Fig. S1 at *JXB* online).

### Gene Ontology enrichment analysis

The AGI codes of the top *A. thaliana* gene accession assigned to each *E. canadensis* gene were used for Gene Ontology (GO) term enrichment analysis. Biological process GO terms that were statistically over-represented (right-sided hypergeometric test, Benjamini–Hochberg correction for multiple testing with α=0.05) in the genes lists consistently differentially expressed among 2,4-D, dicamba, and halauxifen-methyl treatments were determined using the Cytoscape plug-in ClueGO (Bindea *et al.*, 2009). For ontology enrichment, the background reference gene set included homologous *A. thaliana* genes identified in the *E. canadensis* genome that were expressed in leaf tissue. Additional parameters included a minimum of five genes per cluster and a Kappa Score of 0.4.

### Expression analysis validation (qRT–PCR)

To confirm RNA-seq results, five genes identified as differentially expressed from the RNA-seq results were validated with quantitative reverse transcription–PCR (qRT–PCR) at the 1 and 6 HAT time points (Supplementary Table S3). Total RNA was extracted from separate plants sprayed independently from those used for RNA-seq. Leaf tissue was pulverized in a lysis buffer with β-mercaptoethanol, and total RNA was extracted according to the RNeasy Plant Mini Kit protocol with slight modifications (Qiagen cat. no. 74904). To ensure removal of genomic DNA, total RNA was subjected to an RNA Clean & Concentrator kit (Zymo Research cat. no. R1013). cDNA was generated using SuperScript IV reverse transcriptase (Invitrogen cat. no. 18090050) using oligo(dT)_20_ primers (Invitrogen cat. no. 18418-020). Primers were designed using Primer3Plus and prfectBLAST (Santiago-Sotelo and Ramirez-Prado, 2012) (Supplementary Table S3). qRT–PCR was performed in a 10 µl volume and included 5 µl of SYBR green (iQ SYBR Green Supermix, Bio-Rad cat. no. 1708880), 2 µl of 1:1 mix of forward and reverse primers, and 3 µl of 1:30 diluted cDNA. Each reaction was mixed using the QIAgility liquid handling robot (Qiagen cat. no. 9001532) using a custom program. Three technical replicates were performed for each sample, and three biological replicates were performed for each herbicide by time point treatment. The relative fold change of each gene was normalized to a β-tubulin internal control gene (closest Arabidopsis homolog, AT5G23860, *TUB8*). Reaction conditions included one cycle of 3 min at 95 °C, 40 cycles of 15 s at 95 °C, and 30 s at 60 °C, and 1 min at 55 °C. A melt curve analysis confirmed the presence of a single amplified product for each reaction. Relative fold change values were calculated using the 2^−ΔΔCt^ method (Livak and Schmittgen, 2001) with Bioconductor packages NormqPCR and ReadqPCR (Perkins *et al.*, 2012). The qRT–PCR results for each selected gene were consistent with the RNA-seq expression data for each of the three analyses that were performed (Supplementary Fig. S2). The coefficient of determination (*R*^2^) values of 0.767 and 0.751 for DESeq2 and edgeR analysis methods, respectively, indicates a linear relationship between RNA-seq and qRT–PCR results.

### ABA extraction and quantification

Foliar ABA was extracted from (i) leaf tissue collected 1 and 6 HAT; (ii) leaf tissue harvested every 2–3 d after herbicide treatment (DAT) until leaf death; and (iii) leaf tissue harvested from untreated plants from which water was withheld until signs of incipient leaf death. The experimental design included three replications where a replicate represents a unique sprayed or untreated plant. Approximately 0.2 g leaf tissue was collected and immediately weighed (±0.0001 g FW). Fresh leaf tissue was chopped with scissors into a 50 ml centrifuge tube and covered with 8–10 ml of cold (–20 °C) 80% (v/v) methanol in water with added butylated hydroxytoluene. Samples were stored overnight at –20 °C and homogenized the following day. Following homogenization, 15 ng of [^2^H_6_]ABA was added as an internal standard to each tube and samples were stored at 4 °C for 24 h. An aliquot of ~3 ml was removed from each tube, taking care not to disturb the settled pellet, and added to an opaque scintillation vial. Samples were dried to completeness under a vacuum at 37 °C. ABA was resuspended in 120 µl of 2% acetic acid (v/v); the acetic acid solution was used to carefully wash the inside of the vial, taking care not to disturb any settled particulates. All liquid was removed and centrifuged in a separate tube for 3 min at 1500 rpm. A 100 µl aliquot was taken for analysis using an Agilent 6400 series triple quadrupole LC/MS (McAdam and Brodribb, 2018).

### Quantification of leaf water potential

A fully mature leaf from three plants per treatment was carefully excised 1 and 6 HAT and when incipient signs of leaf death were apparent after withholding water with a razor blade and immediately wrapped in a damp paper towel and placed into a sealed plastic bag. Leaf water potential was measured using a Scholander pressure chamber and microscope to accurately determine the balance pressure.

### Quantification of ethylene evolution

Individual plants, including roots and soil, were carefully removed from germination trays and wrapped in aluminum foil to maintain plant moisture and vigor throughout the experiment. These plants were sprayed with 2,4-D, dicamba, halauxifen-methyl, and water as described above. After application, eight treated plants were placed into 20 ml glass vials with metal caps (Agilent, Santa Clara, CA, USA, catalog # 5188-2759) and sealed with a septum (Macherey-Nagel, Bethlehem, PA, USA, catalog #702110). An internal standard of 1 µl of acetylene was manually injected into each treatment vial. At 1, 6, and 24 HAT, a 5 ml sample of gas was taken from each treatment vial and ethylene was measured with a gas chromatograph (Agilent 7890, Agilent Technologies) equipped with a flame ionization detector and a HayeSep N 80/100 mesh silcosteel 1.22 m×1.58 mm column (Agilent Technologies). The injector temperature was 126 °C and helium carrier gas flow was set to 40 ml min^−1^. After analysis, the fresh weight of treated plant material was measured and ethylene evolution was expressed in terms of fresh weight. The experimental design included three replications, where a replicate represents eight treated plants placed into a single vial.

### Statistical analysis

For pairwise comparisons of plant hormone levels and leaf water potential data, Student’s *t*-tests were performed using R version 3.4.1 (R Core Team, Vienna, Austria).

## Results and discussion

**Fig. 1.**
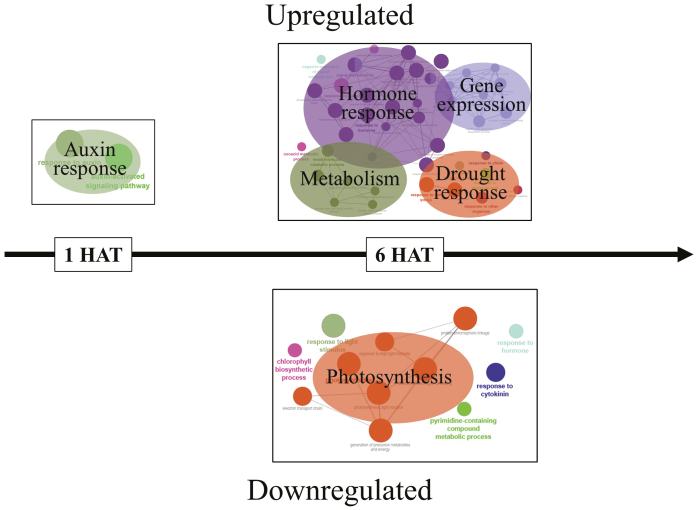
Transcriptomic analysis indicates up-regulation of hormone response genes and the whole-scale down-regulation of genes associated with photosynthesis following auxin herbicide application in *Erigeron canadensis.* Functionally grouped biological process GO terms specific for *E. canadensis* genes up-regulated (top) or down-regulated (bottom) following the application of three synthetic auxin herbicides (2,4-D, dicamba, and halauxifen-methyl) 1 h after herbicide treatment (HAT) and 6 HAT. The node size represents the term enrichment significance. Individual node labels and genes are described in Supplementary Table S4.

Transcriptome sequencing yielded 2.6 billion reads, >99.7% of which passed quality control measures (Supplementary Table S2). Overall mapping rate to the genome ranged from 66% to 77% across all samples. There was a high consistency in the suite of genes differentially expressed following the application of the three auxin herbicides, suggesting that a very similar response pathway was elicited by the three synthetic auxin herbicides (Supplementary Fig. S1; Supplementary Table S3). The enrichment of the GO Biological Process terms associated with the *E. canadensis* genes consistently differentially expressed across the three auxin herbicide treatments at both 1 and 6 HAT illustrates principal clusters of enriched GO terms (Fig. 1). At 1 HAT, the 48 genes up-regulated in all treatments were simplified to a single cluster that included GO terms including the response to auxin and the auxin-activated signaling pathway (Fig. 1). This finding is consistent with other studies that have found that auxin herbicides are perceived by endogenous auxin receptors and the endogenous auxin signaling pathway (Walsh *et al.*, 2006; Gleason *et al.*, 2011).

There were no genes down-regulated by the halauxifen-methyl treatment at 1 HAT and, therefore, no GO term enrichment was conducted for the collective auxin herbicide response for down-regulated genes at that time point. By 6 HAT, canalization in the profile of differentially expressed genes had occurred across the herbicide treatments (Supplementary Fig. S1). The 734 genes up-regulated by the synthetic auxin herbicides at 6 HAT generated the most complex GO term network, although this network yielded only three major clusters: gene expression, hormone signaling, and metabolism (Fig. 1). Analysis of the 516 genes consistently down-regulated by all three auxin herbicides at 6 HAT revealed one major network: photosynthesis (Fig. 1). The down-regulation of the expression of 52 key genes related to photosynthetic processes included genes critical for many key photosynthetic processes. These included genes related to the function of both photosystems, the Calvin cycle, light-harvesting complexes, the electron transport chain, and chlorophyll biosynthesis (Table 1). The primary mode of action of auxin herbicides may not be targeting any particular or specific component of photosynthesis, like the well-known herbicides paraquat (which inhibits electron transfer within PSI) or atrazine (which specifically targets plastoquinone binding in PSII) (Shimabukuro and Swanson, 1969; Dodge and Harris, 1970), but rather may trigger a whole-scale down-regulation of all components of photosynthesis. Whether this whole-scale down-regulation of key genes for photosynthetic function is directly the result of auxin or acts via other plant hormones is a possibility that requires further investigation, but is likely to be a major mechanism by which auxin herbicide application leads to plant death.

**Table 1.** Mean log fold change (logFC) in the expression of *Erigeron canadensis* genes related to photosynthetic processes that were consistently down-regulated in response to the application of three synthetic auxin herbicides (2,4-D, dicamba, and halauxifen-methyl) 6 h after herbicide treatment

*Erigeron canadensis* gene ID	Annotation	BLAST E-value	Arabidopsis	Gene ID	Dicamba	Halauxifen- methyl	2,4-D
**PSI**							
HW_16037_g	PSI subunit E-2	1.69E-40	AT2G20260	*PSAE-2*	–1.60668	–1.64104	–1.4536
HW_48562_g	PSI subunit F	1.70E-111	AT1G31330	*PSAF*	–1.16121	–1.26456	–0.98346
HW_9242_g	PSI subunit H-1	2.05E-70	AT3G16140	*PSAH-1*	–1.17022	–1.29681	–1.08693
HW_33889_g	PSI subunit l	5.87E-104	AT4G12800	*PSAL*	–0.91515	–0.96488	–0.77821
HW_31687_g	PSI reaction center subunit PSI-N, chloroplast, putative/PSI-N, putative (PSAN)	5.36E-73	AT5G64040	*PSAN*	–1.19443	–1.45038	–1.09003
HW_10539_g	PSI type III Chl *a*/*b*-binding protein	7.02E-163	AT1G61520	*LHCA3*	–2.22354	–2.30984	–1.9535
HW_42835_g	PSI type III Chl *a*/*b*-binding protein	1.62E-161	AT1G61520	*LHCA3*	–0.89255	–1.13674	–0.87659
HW_4678_g	Light-harvesting chlorophyll–protein complex II subunit B1	3.81E-74	AT2G34430	*LHB1B1*	–5.64799	–4.75468	–5.45167
HW_47482_g	Light-harvesting chlorophyll–protein complex I subunit A4	2.79E-152	AT3G47470	*LHCA4*	–1.82566	–2.00102	–1.83264
HW_24247_g	Rhodanese/cell cycle control phosphatase superfamily protein	1.78E-48	AT2G42220		–1.20809	–1.5702	–1.43429
HW_34450_g	Rhodanese/cell cycle control phosphatase superfamily protein	1.63E-81	AT3G08920		–0.90287	–1.22676	–1.20918
HW_8257_g	Thylakoid rhodanese-like protein	1.23E-130	AT4G01050	*TROL*	–1.09777	–1.34657	–1.06723
**PSII**							
HW_3815_g	PSII reaction center PSB28 protein	7.92E-67	AT4G28660	*PSB28*	–0.72277	–1.19023	–0.66968
HW_34248_g	PSII subunit O-2	0.00E+00	AT3G50820	*PSBO2*	–1.10818	–1.30558	–1.06907
HW_52495_g	PSII subunit P-1	1.95E-103	AT1G06680	*PSBP-1*	–1.00943	–1.21259	–0.89012
HW_49334_g	PSII subunit Q-2	1.36E-106	AT4G05180	*PSBQ-2*	–1.08268	–1.28959	–1.06212
HW_44270_g	PSII light-harvesting complex protein 2.1	1.77E-173	AT2G05100	*LHCB2.1*	–1.73548	–1.92219	–1.38791
HW_44269_g	PSII light-harvesting complex protein 2.2	2.82E-179	AT2G05070	*LHCB2.2*	–2.29512	–2.24142	–1.91361
HW_39414_g	Light-harvesting complex PSII	9.81E-157	AT3G08940	*LHCB4.2*	–1.15774	–1.16205	–0.87397
HW_53979_g	Light-harvesting complex of PSII 5	1.32E-59	AT4G10340	*LHCB5*	–2.08755	–1.95895	–1.51739
HW_13989_g	Light-harvesting complex of PSII 5	1.23E-121	AT4G10340	*LHCB5*	–1.13005	–1.73818	–1.36219
HW_48222_g	Light-harvesting complex of PSII 5	5.44E-152	AT4G10340	*LHCB5*	–1.2222	–1.4342	–1.25446
HW_28848_g	Serine/threonine-kinase pakA-like protein	3.12E-46	AT3G15095	*HCF243*	–3.02674	–2.32637	–2.8792
HW_6455_g	Chl *a*/*b*-binding protein 2	2.22E-167	AT1G29920	*CAB2*	–3.416	–3.24033	–2.81991
HW_36583_g	Chl *a*/*b*-binding protein 2	3.02E-158	AT1G29920	*CAB2*	–3.012	–2.92871	–2.67951
HW_46472_g	Light-harvesting Chl *b*-binding protein 3	1.42E-176	AT5G54270	*LHCB3*	–3.416	–3.24033	–2.81991
HW_26094_g	Light-harvesting complex PSII subunit 6	6.32E-151	AT1G15820	*LHCB6*	–1.68159	–1.53985	–1.19332
**Electron transport**							
HW_30946_g	2Fe–2S ferredoxin-like superfamily protein	2.10E-57	AT1G60950	*FED A*	–1.62827	–1.83487	–1.61536
HW_30945_g	2Fe–2S ferredoxin-like superfamily protein	1.75E-40	AT1G60950	*FED A*	–1.5311	–1.55179	–1.36328
HW_30799_g	High cyclic electron flow 1	0.00E+00	AT3G54050	*HCEF1*	–1.31364	–1.79291	–1.47226
HW_46113_g	Photosynthetic electron transfer C	3.20E-112	AT4G03280	*PETC*	–0.83696	–0.91175	–0.64561
HW_7585_g	NDH-dependent cyclic electron flow 1	4.43E-81	AT3G16250	*PnsB3*	–1.34119	–1.69163	–1.60495
HW_6988_g	2Fe–2S ferredoxin-like superfamily protein	3.01E-34	AT4G14890	*FdC1*	–1.00388	–1.06538	–0.80869
HW_10771_g	Pyridine nucleotide-disulfide oxidoreductase family protein	0.00E+00	AT1G74470		–1.23119	–1.66043	–0.95949
HW_42602_g	Protein containing PDZ domain, a K-box domain, and a TPR region	5.83E-147	AT1G55480	*ZKT*	–0.81759	–1.36575	–1.08815
**ATP and carbohydrate synthesis**							
HW_48988_g	F-type H-transporting ATPase subunit delta	2.12E-89	AT4G09650	*ATPD*	–1.09461	–1.31901	–1.27576
HW_715_g	ATPase, F1 complex, gamma subunit protein	0.00E+00	AT4G04640	*ATPC1*	–0.67819	–0.9783	–0.76126
HW_51130_g	ADP glucose pyrophosphorylase 1	0.00E+00	AT5G48300	*ADG1*	–1.04462	–1.49256	–1.22244
HW_12082_g	Pyruvate kinase family protein	4.04E-118	AT3G52990		–0.96522	–1.30784	–1.09865
HW_36948_g	Glycosyl transferase, family 35	0.00E+00	AT3G29320	*PHS1*	–0.90018	–1.01497	–1.38361
**Calvin cycle**							
HW_31087_g	Phosphoribulokinase	0.00E+00	AT1G32060	*PRK*	–0.91459	–1.42335	–1.4587
HW_32431_g	Rubisco (small chain) family protein	2.05E-103	AT5G38430	*RBCS1B*	–2.11033	–1.73922	–1.17414
HW_20259_g	Rubisco (small chain) family protein	1.09E-102	AT5G38430	*RBCS1B*	–0.93894	–0.96644	–0.54165
HW_15482_g	Sedoheptulose-bisphosphatase	0.00E+00	AT3G55800	*SBPASE*	–1.21224	–1.61282	–1.38584
HW_3712_g	Fructose-bisphosphate aldolase 2	0.00E+00	AT4G38970	*FBA2*	–1.7884	–2.27548	–2.07169
**Carbon concentrating**							
HW_34543_g	alpha-Carbonic anhydrase 1	2.16E-76	AT3G52720	*CA1*	–1.97886	–1.64024	–2.01499
**Thermal tolerance**							
HW_2391_g	Fatty acid desaturase 5	8.64E-107	AT3G15850	*FAD5*	–1.52487	–2.2455	–2.30328
HW_49631_g	Fatty acid desaturase 5	8.97E-96	AT3G15850	*FAD5*	–1.75132	–1.68668	–2.11965
**Chlorophyll synthesis**							
HW_46247_g	Magnesium-chelatase subunit chlH, chloroplast, putative/Mg-protoporphyrin IX chelatase, putative (CHLH)	0.00E+00	AT5G13630	*GUN5*	–1.43737	–1.74361	–1.30458
**Gene expression**							
HW_20063_g	Sigma factor A	0.00E+00	AT1G64860	*SIGA*	–1.93346	–2.14484	–1.85543
HW_25442_g	Ribosomal protein L23AB	5.08E-22	AT3G55280	*RPL23AB*	–0.82058	–1.10878	–0.86017

The unique *Erigeron canadensis* gene ID and annotation based on the most closely associated Arabidopsis gene identified by BLAST is presented

**Fig. 2.**
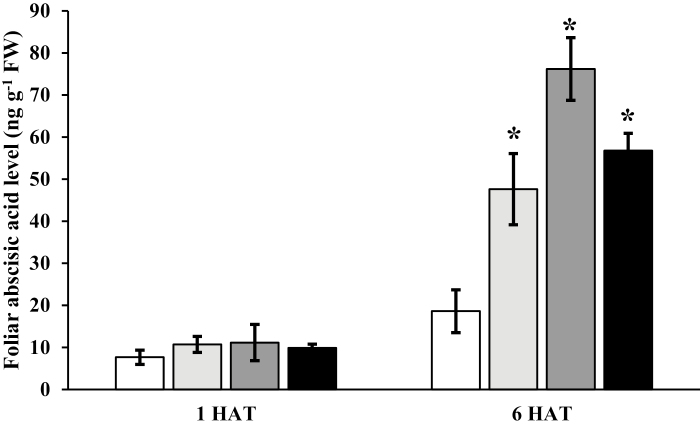
Considerable production of foliar abscisic acid (ABA) after auxin herbicide application in *Erigeron canadensis*. Foliar ABA levels in *E. canadensis* leaves 1 h and 6 h after treatment (HAT) with water (white) and three synthetic auxin herbicides [2,4-D (light gray), dicamba (dark gray), or halauxifen-methyl (black)]. Data represent the mean of three replicates ±SE. An asterisk denotes a significant difference in value compared with the water control within (*P*<0.05).

At 1 and 6 HAT, the *E. canadensis* homolog of *A. thaliana NCED3* was consistently up-regulated by all three synthetic auxin herbicides (Supplementary Table S5). In contrast, no significant differential expression in any major ethylene biosynthesis genes, including *ACS*, was observed at 1 HAT and only a slight increase was observed in *ACS6* at 6 HAT (Supplementary Table S6). Reflecting a minimal impact of auxin herbicides on ethylene homeostasis in *E. canadensis*, there was an inconsistent increase in ethylene levels across herbicides, with a significant change observed in response to the application of 2,4-D and dicamba, at 24 HAT. Consistent up-regulation of *NCED* across all synthetic herbicide treatments relative to the control at 1 and 6 HAT was confirmed by qRT–PCR (Supplementary Table S5). Despite measured increases in the expression of *NCED*, there was no change in ABA level at 1 HAT (Fig. 2), although at 6 HAT there was a significant increase in ABA levels in the treated leaves compared with the control (Fig. 2). The dicamba treatment resulted in the highest ABA level of 76 ng g^−1^, while plants treated with 2,4-D and halauxifen-methyl accumulated 47 ng g^−1^ and 56 ng g^−1^ of ABA, respectively. The delay in ABA accumulation following increased *NCED* expression may indicate a lag because of limited expression in the genes responsible for catalyzing the downstream steps in the ABA biosynthetic pathway. This increase in *NCED* expression and ABA levels is similar to that reported for *G. aparine* following herbicide treatment (Kraft *et al.*, 2007).

**Fig. 3.**
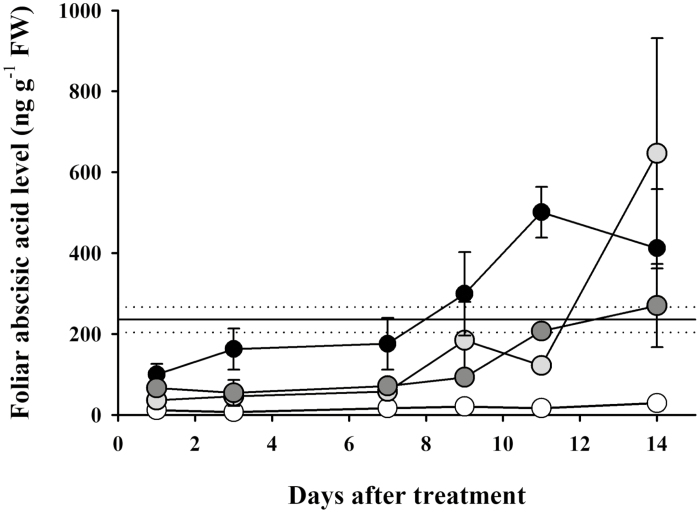
Continual production of foliar abscisic acid (ABA) until leaf death after auxin herbicide application in *Erigeron canadensis*. Foliar ABA levels in *E. canadensis* measured after treatment with water (white) or three synthetic auxin herbicides [2,4-D (light gray), dicamba (dark gray), or halauxifen-methyl (black)], until leaves showed signs of necrosis. The horizontal solid line depicts mean foliar ABA levels (dotted lines depict the upper and lower bounds of the SE) in water-stressed *E. canadensis* plants at the point of incipient leaf death (mean leaf water potential: –1.2 MPa). Data represent the mean of three replicates ±SE.

During drought, increased ABA levels promote plant survival by closing stomata, reducing transpiration, as well as activating a suite of desiccation tolerance genetic pathways. In this study, ABA levels continued to increase in leaves following auxin herbicide application until the leaves began displaying regions of necrosis 14 d after application (Fig. 3). In all herbicide treatments, mean ABA levels 14 d after application were between 270 ng g^−1^ and 650 ng g^−1^ FW (Fig. 3). In drought-stressed plants in which water had been withheld until incipient signs of leaf death (mean leaf water potential: –1.2 MPa), mean foliar ABA levels were 235 ng g^−1^ (Fig. 3). The nearly 3-fold higher levels of foliar ABA in auxin herbicide-treated leaves at the point of leaf death compared with water-stressed leaves suggests that ABA level homeostasis was severely altered by the synthetic auxin herbicides. ABA has been found to play an important role in triggering leaf senescence (Lim *et al.*, 2007). Recent work by Zhao *et al.* (2016) showed that ABA-triggered leaf senescence is an ethylene-independent process, with ABA perception mutants in an ABA receptor gene as well as downstream proteins in the ABA signaling pathway having strong non-senescent phenotypes. The rapid synthesis and dramatic accumulation of ABA, exceeding that of drought-stressed leaves, following auxin herbicide application may have contributed to the rapid down-regulation of genes related to photosynthesis via this SnRK2 senescence pathway and perhaps to the death of leaves 2 weeks after herbicide application.

**Fig. 4.**
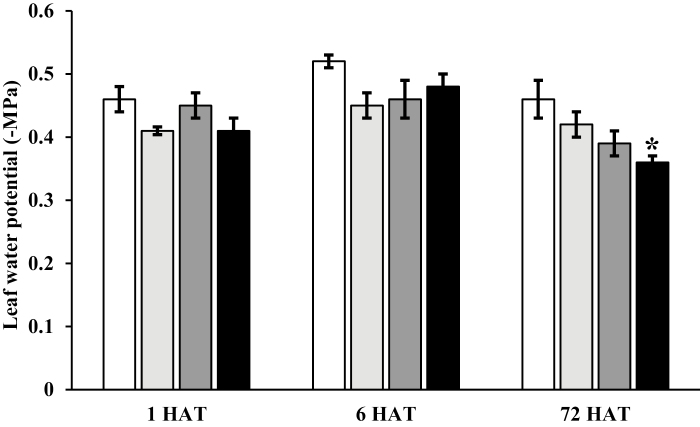
No significant change in leaf water potential after auxin herbicide application in *Erigeron canadensis*. Leaf water potential in *E. canadensis* leaves 1, 6, and 72 h after treatment (HAT) with water (white) and three synthetic auxin herbicides [2,4-D (light gray), dicamba (dark gray), or halauxifen-methyl (black)]. Data represent the mean of three replicates ±SE. An asterisk denotes a significant difference compared with water (control) (*P*<0.05).

Up-regulation of *NCED* in leaves is known to be driven by a decline in leaf water status leading to a loss in cell turgor or cell volume (McAdam *et al.*, 2016; Sussmilch *et al.*, 2017). Thus, treatment with synthetic auxin herbicides may have caused a change in cell turgor pressure triggering the up-regulation of *NCED*. However, there was no consistent reduction in leaf water potential at 1, 6, or 72 HAT in *E. canadensis* after synthetic auxin herbicide application (Fig. 4). At 72 HAT, leaf water potential was less negative following halauxifen-methyl treatment compared with control plants, suggesting that plants had rehydrated, which might be due to stomatal closure caused by the increase in ABA levels. These results suggest that the auxin-induced increase in *NCED* expression is independent of reductions in leaf turgor, which is the main trigger for *NCED* expression and ABA biosynthesis at high VPD or during soil drought (Qin and Zeevaart, 1999; McAdam *et al.*, 2016).

**Fig. 5.**
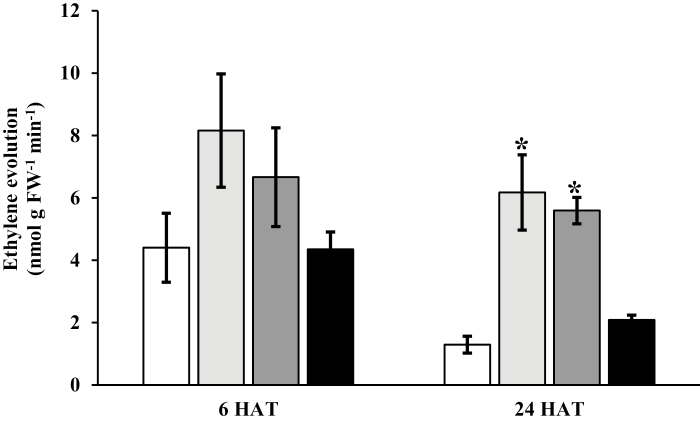
Inconsistent and slow ethylene production after auxin herbicide application in *Erigeron canadensis*. Ethylene evolution from *E. canadensis* leaves 6 h and 24 h after treatment (HAT) with water (white) and three synthetic auxin herbicides [2,4-D (light gray), dicamba (dark gray), or halauxifen-methyl (black)]. Data represent the mean of three replicates ±SE. An asterisk denotes a significant difference compared with water (control) (*P*<0.05). The ethylene level was undetectable in any of the samples at 1 HAT so that time point is not included in the figure.

In agreement with the transcriptomic data which showed only minimal increases in one ethylene biosynthesis gene at 6 HAT, there was no significant increase in ethylene evolution at 6 HAT from *E. canadensis* rosettes treated with 2,4-D, dicamba, or halauxifen-methyl compared with the control, although by 24 HAT there was a very limited yet significant increase in ethylene levels in the plants treated with 2,4-D and dicamba (Fig. 5). This result contrasts with the findings of Kraft *et al.* (2007) in *G. aparine*, which displayed a substantial increase in ethylene evolution within 2 HAT. At 24 HAT, *E. canadensis* rosettes treated with 2,4-D and dicamba produced 6.2 nmol ethylene g^−1^ min^−1^ and 5.6 nmol ethylene g^−1^ min^−1^, respectively, compared with 1.3 nmol ethylene g^−1^ min^−1^ produced in the control plants (Fig. 5). However, there was no increase in ethylene evolution following the foliar application of halauxifen-methyl; this result suggests a possible differential response of *E. canadensis* to the unique arylpicolinate chemistry of halauxifen-methyl compared with 2,4-D and dicamba. These results suggest that ethylene may not be a primary and essential component of auxin herbicide-induced plant death as proposed by Kraft *et al.* (2007). The consistent response across three species observed to date (*E. canadensis*, *A. thaliana*, and *G. aparine*) of activation of ABA biosynthesis and an increase in ABA levels suggests that if any other phytohormone is playing a role in the induction of plant death in response to synthetic auxin herbicide application it is ABA.

Using a transcriptomic approach to investigate the mode of action of auxin herbicides, we find that synthetic auxin herbicides down-regulate a suite of genes related to photosynthetic processes, rather than targeting an individual component of photosynthesis. This whole-scale down-regulation of the photosynthetic process may be the primary driver behind plant death in plants treated with auxin herbicides, which generally survive in a state of suspended, or compromised, growth for some time before death (Sterling and Hall, 1997). The down-regulation of photosynthesis in response to auxin herbicide application is not due to an increase in ethylene levels, as has been proposed by Kraft *et al.* (2007), with the levels of this hormone remaining unchanged following auxin herbicide application in *E. canadensis*. Auxin herbicides consistently up-regulate ABA biosynthesis, and this up-regulation is independent of a change in leaf water status. The whole-scale down-regulation of genes related to photosynthesis in response to auxin herbicides may be due to the action of both auxin and ABA.

## Supplementary data

Supplementary data are available at *JXB* online.

Fig. S1. Venn diagrams illustrating differentially expressed genes in *Erigeron canadensis* following synthetic auxin herbicide application.

Fig. S2. Scatter plots of relative gene expression measured by qRT–PCR versus estimation from RNA-seq.

Table S1. Transcriptomic quality control and mapping statistics for *Erigeron canadensis*.

Table S2. Number of differentially expressed genes for each analysis method.

Table S3. Primer sequences used for qRT–PCR validation of gene expression in *Erigeron canadensis*.

Table S4. Biological process GO terms specific for *Erigeron canadensis* genes regulated by auxin herbicides.

Table S5. Relative expression levels of 9-*cis*-epoxycarotenoid dioxygenase (*NCED*) in *Erigeron canadensis* leaves

Table S6. Differentially expressed genes discussed in the text.

eraa124_suppl_Supplementary_Material

## Data Availability

RNA-seq data are accessible through GEO Series accession number GSE116958 (https://www.ncbi.nlm.nih.gov/geo/query/acc.cgi?&acc=GSE116958).
