# Supplementary material for: Transcriptomics in Erigeron canadensis reveals rapid photosynthetic and hormonal responses to auxin herbicide application
Source: J Exp Bot. 2020 Mar 12;71(12):3701–9. doi: 10.1093/jxb/eraa124 (PMC7307852; doi:10.1093/jxb/eraa124)
Supplement: eraa124_suppl_Supplementary_Material [file eraa124_suppl_supplementary_material.pdf]

Transcriptomics in *Erigeron canadensis* reveals rapid photosynthetic and hormonal responses to auxin-herbicide application

Authors: Cara L. McCauley, Scott A.M. McAdam, Ketaki Bhide, Jyothi Thimmapuram, Jo Ann Banks, Bryan G. Young

The following Supplemental Information is available for this article:

Fig. S1 Venn diagrams illustrating differentially expressed genes in horseweed following synthetic auxin herbicide application. Increase or decrease in expression was determined by three analysis methods: edgeR, DESeq2, and Cufflinks; pairwise comparisons were made between each herbicide treatment and the water (control) treatment at the same time point. Genes that were significantly differentially expressed (adjP ≤ 0.05) in at least two of the three analysis methods are shown. A. upregulated 1 HAT; B. downregulated 1 HAT; C. upregulated 6 HAT; D. downregulated 6 HAT.

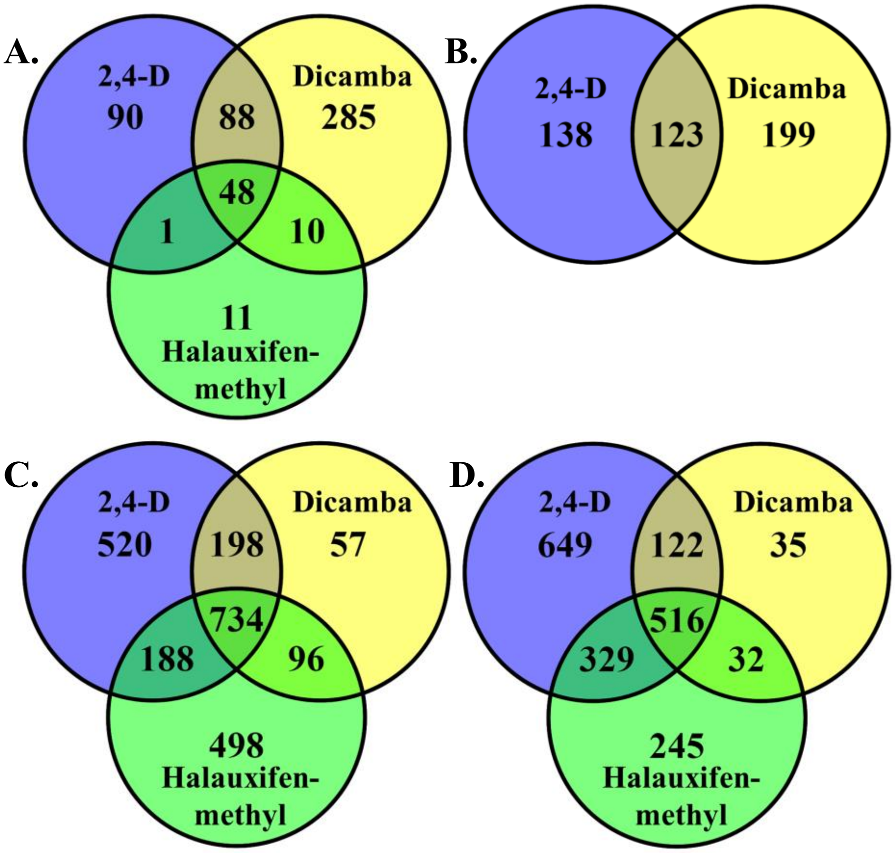

Fig. S2 Scatter plots of relative expression measured by qRT-PCR versus estimation from RNA-seq. Each plot represents five genes measured following three herbicide treatments at two time points; 30 unique points of comparison for each of the differential gene expression analysis methods is shown with the corresponding R^2 value; A. DESeq2, B. edgeR, C. Cufflinks.

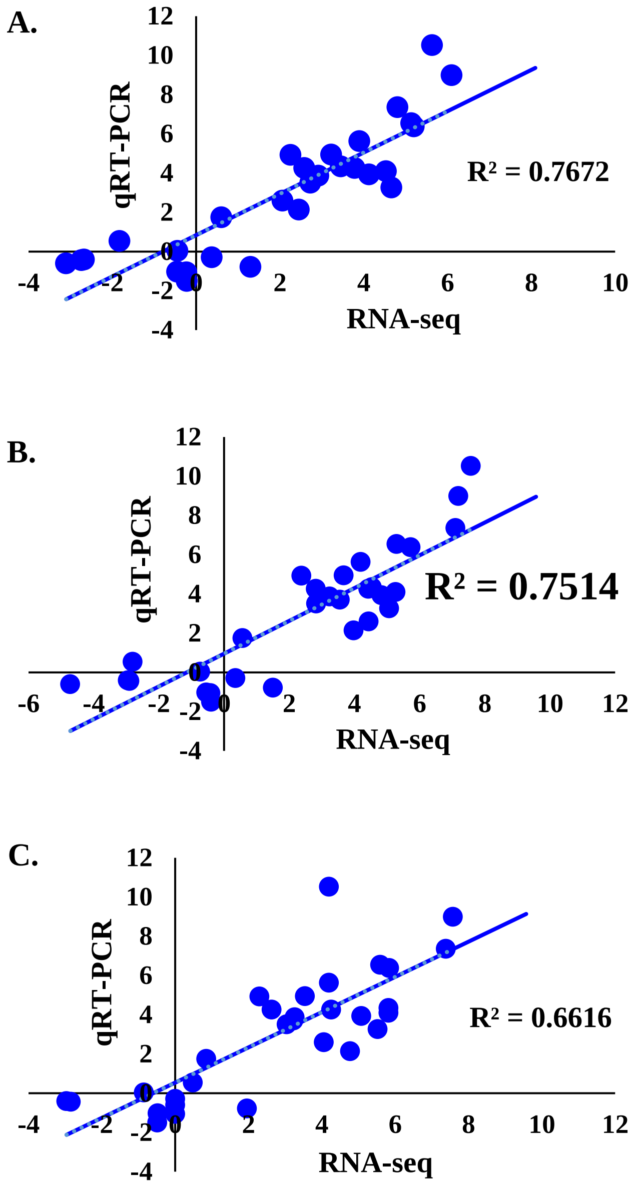

Table S1 Quality control and mapping statistics for *Erigeron canadensis*.

Sample ID	Total PE Reads	QC passed PE Reads	Reads Used In Mapping	Total Reads Mapped	Overall Mapping Rate	% Mapped Reads Assigned to Genes
DE1_1.fastq	48,214,670	48,195,783	96,251,306	67,900,186	70.54	31.04
DE1_2.fastq	48,214,670	48,142,446				
DE2_1.fastq	43,277,927	43,259,528	86,423,406	63,994,484	74.05	31.35
DE2_2.fastq	43,277,927	43,228,701				
DE3_1.fastq	46,245,538	46,221,333	92,305,360	70,485,957	76.36	30.08
DE3_2.fastq	46,245,538	46,174,177				
DE4_1.fastq	40,937,445	40,924,130	81,759,876	58,784,626	71.90	31.07
DE4_2.fastq	40,937,445	40,891,048				
DL1_1.fastq	36,750,941	36,739,168	73,367,844	55,073,742	75.07	30.45
DL1_2.fastq	36,750,941	36,694,571				
DL2_1.fastq	45,944,049	45,904,534	91,647,472	68,201,069	74.42	28.57
DL2_2.fastq	45,944,049	45,852,831				
DL3_1.fastq	33,712,261	33,698,247	67,268,020	44,881,599	66.72	31.54
DL3_2.fastq	33,712,261	33,647,698				
DL4_1.fastq	43,257,202	43,245,271	86,377,348	64,803,622	75.02	30.00
DL4_2.fastq	43,257,202	43,199,798				
HE1_1.fastq	31,370,071	31,362,497	62,679,086	47,964,458	76.52	30.39
HE1_2.fastq	31,370,071	31,346,121				
HE2_1.fastq	47,264,931	47,242,757	94,386,336	71,104,756	75.33	29.99
HE2_2.fastq	47,264,931	47,213,019				
HE3_1.fastq	43,647,456	43,626,646	87,142,850	65,442,194	75.10	30.31
HE3_2.fastq	43,647,456	43,589,885				
HE4_1.fastq	41,328,752	41,304,222	82,470,648	59,913,818	72.65	30.10
HE4_2.fastq	41,328,752	41,257,517				
HL1_1.fastq	41,908,704	41,894,263	83,713,276	61,798,858	73.82	30.39
HL1_2.fastq	41,908,704	41,869,901				
HL2_1.fastq	55,535,109	55,518,049	110,909,004	82,807,785	74.66	28.24
HL2_2.fastq	55,535,109	55,470,768				
HL3_1.fastq	30,771,275	30,758,131	61,430,866	45,770,753	74.51	30.28
HL3_2.fastq	30,771,275	30,728,167				
HL4_1.fastq	40,337,486	40,314,640	80,498,232	60,851,780	75.59	29.70
HL4_2.fastq	40,337,486	40,270,945				
TE1_1.fastq	42,856,301	42,844,152	85,609,214	63,355,770	74.01	31.40
TE1_2.fastq	42,856,301	42,813,824				
TE2_1.fastq	47,216,774	47,198,429	94,279,974	66,453,692	70.49	31.48
TE2_2.fastq	47,216,774	47,154,948				
TE3_1.fastq	61,895,157	61,870,735	123,608,220	93,860,612	75.93	31.02

TE3_2.fastq	61,895,157	61,826,528				
TE4_1.fastq	34,971,467	34,952,857	69,780,124	50,650,190	72.59	31.36
TE4_2.fastq	34,971,467	34,907,454				
TL1_1.fastq	36,283,515	36,273,380	72,453,930	53,989,880	74.52	30.87
TL1_2.fastq	36,283,515	36,236,412				
TL2_1.fastq	43,398,458	43,373,513	86,602,532	62,598,349	72.28	30.65
TL2_2.fastq	43,398,458	43,319,193				
TL3_1.fastq	37,693,393	37,668,560	75,165,156	54,085,558	71.96	31.28
TL3_2.fastq	37,693,393	37,604,735				
TL4_1.fastq	39,027,020	38,998,574	77,809,878	56,050,438	72.04	30.71
TL4_2.fastq	39,027,020	38,928,043				
WE1_1.fastq	37,332,228	37,320,976	74,563,720	55,512,211	74.45	31.20
WE1_2.fastq	37,332,228	37,292,177				
WE2_1.fastq	31,504,072	31,487,603	62,905,670	46,789,742	74.38	30.79
WE2_2.fastq	31,504,072	31,467,298				
WE3_1.fastq	27,427,218	27,416,216	54,778,264	40,935,813	74.73	30.75
WE3_2.fastq	27,427,218	27,398,591				
WE4_1.fastq	33,345,272	33,333,002	66,613,656	51,516,544	77.34	30.49
WE4_2.fastq	33,345,272	33,318,294				
WL1_1.fastq	41,809,361	41,795,883	83,456,836	63,378,573	75.94	29.95
WL1_2.fastq	41,809,361	41,740,045				
WL2_1.fastq	45,085,407	45,065,659	90,026,164	68,256,344	75.82	30.23
WL2_2.fastq	45,085,407	45,029,555				
WL3_1.fastq	37,799,578	37,782,739	75,455,092	56,810,796	75.29	29.65
WL3_2.fastq	37,799,578	37,742,449				
WL4_1.fastq	34,881,233	34,871,233	69,678,914	52,610,248	75.50	31.05
WL4_2.fastq	34,881,233	34,848,727				

Table S2 Number of differentially expressed genes (adjP ≤ 0.05) from each analysis method.

Time point	Herbicide treatment	DESeq2	edgeR	Cufflinks	# genes detected by 2 or more methods
1 HAT	2,4-D	736	381	725	488
	Dicamba	1,327	610	1121	753
	Halauxifen-methyl	110	60	147	70
6 HAT	2,4-D	3,797	2,885	3,378	3,256
	Dicamba	2,352	1,593	1,813	1,790
	Halauxifen-methyl	3,272	2,219	2,820	2,638

Table S3 Primer sequences used for qRT-PCR validation of gene expression in horseweed.

Horseweed gene	Annotation	Forward sequence (5' to 3')	Reverse sequence (5' to 3')
hw34273	NCED5	TCGACCGGTTTTCCCAAAAG	ACCAAACCGAACAACGAACG
hw1898	Cinnamyl alcohol dehydrogenase	TTTTGGCGCGGAAGTTACTG	ATCCAATGAACTCGCTGCAG
hw28000	Starch synthase 2	AGCTAATCCATGCACTTGGC	TGCATCATTCCACGTTGCTG
hw18003	SAUR-like	TCAAGAAGAGTACGGCTTCACC	TCCTTTGCACATCGAAGCAC
hw53227	GH3.1	TTTCAAGACACGGGCCTTTG	ATTGCACCAAGACGAAGCAC
hw21538	Tubulin	ACGCTACCTAACTGCATCTGTC	AGGGATGTCACAAACGCTTG

Table S4 Biological process GO terms specific for horseweed genes consistently regulated by 2,4-D, dicamba, and halauxifen-methyl at 1 and 6 HAT. There were no downregulated terms at 1 HAT.

[a] Benjamini-Hochberg adjusted p-value (α=0.05).
[b] Percentage of genes mapped to the GO term compared to all genes associated with the term.

GOID	GOTerm	Adjusted P-value[a]	Genes (%)[b]	Genes (#)	Associated Genes
Upregulated at 1 HAT					
GO:0009733	response to auxin	0.000000	7	11	[ARF16, AT1G69160, AT2G21210, AT3G13980, AT4G38840, ATAUX2-11, GH3.1, IAA13, IAA29, SAUR22, SHY2]
GO:0009734	auxin-activated signaling pathway	0.000000	10	8	[ARF16, AT1G69160, AT3G13980, ATAUX2-11, IAA13, IAA29, SAUR22, SHY2]
Upregulated at 6 HAT					
GO:0009725	response to hormone	0.000000	15	110	[AAE3, AATP1, ABCG40, ABF2, ABF4, ACC1, ACO3, ACS6, ALDH7B4, APS2, ARF19, AT1G62660, AT1G69160, AT1G75580, AT2G17500, AT2G21210, AT2G25070, AT2G30020, AT2G37030, AT2G44060, AT3G12955, AT3G13980, AT3G51450, AT4G13620, AT4G38840, AT5G14920, AT5G52020, AT5G61890,

					ATAF1, ATAF2, ATAUX2-11, BLH1, BRH1, BSK1, BSK5, BSL1, BZR1, C2, CBL9, CP1, CPK5, CRF10, CRF4, CTR1, ChiC, D6PKL2, DEAR2, DFL1, DOR, EBF2, EIN2, ERD10, ERF-1, ERF1, ERF13, ERF9, ESE3, ETR2, ETT, EXO, FER, GA3OX1, GH3.1, GP ALPHA 1, HAI2, HB-2, HB-7, HK3, HK5, IAA13, IAA29, IAA9, ILL6, IP5PII, JAZ1, JAZ10, JAZ6, LAX2, MAKR6, MYB15, MYB73, MYB77, MYB78, NAC083, PAD4, PAP1, PIF3, PIN4, PRE5, RAN1, RAP2.7, RAV1, RAX2, RD26, RHM1, Rap2.6L, SHY2, SK32, STZ, TDR1, THFS, TIFY10B, TLP1, TPL, TPS10, TT4, WES1, WRKY6, YLS2, ZFP7]
GO:0009755	hormone-mediated signaling pathway	0.000000	19	69	[ABF2, ABF4, ARF19, AT1G62660, AT1G69160, AT2G17500, AT2G30020, AT3G13980, AT4G13620, AT5G14920, AT5G52020, AT5G61890, ATAF1, ATAUX2-11, BSK1, BSK5, BSL1, BZR1, C2, CBL9, CPK5, CRF10, CRF4, CTR1, D6PKL2, DEAR2, DFL1, DOR, EBF2, EIN2, ERF-1, ERF1, ERF13, ERF9, ESE3, ETR2, ETT, FER, GA3OX1, GP ALPHA 1, HAI2, HB-7, HK3, HK5, IAA13, IAA29, IAA9, IP5PII, JAZ1, JAZ10, JAZ6, LAX2, MAKR6, PAD4, PAP1, PIF3, PIN4, PRE5, RAN1, RAP2.7, RAV1, RHM1, Rap2.6L, SHY2, TDR1, TIFY10B, TPL, WRKY6, ZFP7]
GO:0010033	response to organic substance	0.000000	13	120	[AAE3, AATP1, ABCG40, ABF2, ABF4, ACC1, ACO3, ACS6, ALDH7B4, APS2, ARF19, AT1G62660, AT1G69160, AT1G75580, AT2G17500, AT2G21210, AT2G25070, AT2G30020, AT2G37030, AT2G44060, AT3G12955, AT3G13980, AT3G51450, AT4G13620, AT4G38840, AT5G14920,

					AT5G52020, AT5G61890, ATAF1, ATAF2, ATAUX2-11, BLH1, BRH1, BSK1, BSK5, BSL1, BZR1, C2, CBL9, CP1, CPK5, CRF10, CRF4, CTR1, ChiC, D6PKL2, DEAR2, DFL1, DMR6, DOR, EBF2, EFE, EIN2, ERD10, ERF-1, ERF1, ERF13, ERF9, ESE3, ETR2, ETT, EXO, FER, GA3OX1, GH3.1, GP ALPHA 1, HAI2, HB-2, HB-7, HK3, HK5, IAA13, IAA29, IAA9, ILL6, IP5PII, JAZ1, JAZ10, JAZ6, KTI1, LAX2, MAKR6, MEKK1, MYB15, MYB73, MYB77, MYB78, NAC083, NPH3, OPR1, PAD4, PAP1, PIF3, PIN4, PRE5, RAN1, RAP2.7, RAV1, RAX2, RD26, RHM1, RING1, Rap2.6L, SHY2, SK32, STZ, SUS3, TDR1, THFS, TIFY10B, TLP1, TPL, TPS10, TT4, WAK2, WES1, WRKY33, WRKY6, YLS2, ZFP7]
GO:1901700	response to oxygen-containing compound	0.000000	14	105	[AATP1, ABCG40, ABF2, ABF4, ACO3, ACS6, ALDH7B4, AT1G62660, AT2G21210, AT2G25070, AT2G30020, AT2G44060, AT3G17800, AT3G51450, AT5G14920, ATAF1, ATAF2, BLH1, BRH1, BSK1, BSK5, BSL1, BZR1, C2, CAMBP25, CBL9, CCD1, CIPK23, CP1, CPK5, CRK2, CTR1, ChiC, DMR6, DOR, EFE, EGY3, EIN2, ERD10, ERD15, ERF-1, ERF1, ERF13, EXO, FER, FER4, GA3OX1, GP ALPHA 1, HAI2, HB-7, HK3, HK5, HSP70, ILL6, IP5PII, JAZ1, JAZ10, JAZ6, KT1, KTI1, LACS7, MAKR6, MEKK1, MGL, MYB15, MYB73, MYB77, MYB78, NAC017, NAC083, NCED5, NRT1.5, NRT2.7, OPR1, OPR3, PAD4, PAL1, PAP1, PIF3, PRE5, RAV1, RAX2, RD19, RD2, RD26, RING1, Rap2.6L, SIP3, SK32, SLAH3, STZ, SUS3, TDR1, TIFY10B, TPL,

					TPS10, TT4, WAK2, WR3, WRKY33, WRKY6, XDH1, YLS2, ZFP7, ZIFL1]
GO:0001101	response to acid chemical	0.000000	15	87	[AATP1, ABCG40, ABF2, ABF4, ACO3, ACS6, ALDH7B4, AT1G62660, AT2G25070, AT2G30020, AT2G44060, AT3G17800, AT3G51450, AT5G14920, ATAF1, ATAF2, BLH1, BSK1, C2, CAMBP25, CBL9, CCD1, CIPK23, CP1, CPK5, CRK2, ChiC, DMR6, DOR, EFE, EIN2, ERD10, ERD15, ERF1, FER, GA3OX1, GP ALPHA 1, HAI2, HB-7, HK3, HK5, ILL6, IP5PII, JAZ1, JAZ10, JAZ6, KT1, KTI1, LACS7, MAKR6, MEKK1, MGL, MYB15, MYB73, MYB77, MYB78, NAC083, NCED5, NRT1.5, NRT2.7, OPR1, OPR3, PAD4, PAL1, PAP1, PIF3, PRE5, RAX2, RD19, RD2, RD26, Rap2.6L, SIP3, SLAH3, STZ, SUS3, TIFY10B, TPL, TPS10, TT4, WAK2, WR3, WRKY33, XDH1, YLS2, ZFP7, ZIFL1]
GO:0009723	response to ethylene	0.000000	27	35	[ABCG40, ACS6, APS2, ARF19, AT3G51450, AT4G13620, AT5G52020, AT5G61890, CRF10, CRF4, CTR1, DEAR2, EBF2, EIN2, ERF-1, ERF1, ERF13, ERF9, ESE3, ETR2, FER, HK5, MYB15, MYB73, MYB77, PAD4, PAP1, RAN1, RAP2.7, RAV1, Rap2.6L, TDR1, TLP1, WRKY6, YLS2]
GO:0070887	cellular response to chemical stimulus	0.000000	14	77	[ABF2, ABF4, ACS6, ARF19, AT1G62660, AT1G69160, AT2G17500, AT2G30020, AT3G13980, AT4G13620, AT5G14920, AT5G52020, AT5G61890, ATAF1, ATAUX2-11, BOR4, BSK1, BSK5, BSL1, BZR1, C2, CBL9, CPK5, CRF10, CRF4, CTR1, D6PKL2, DEAR2, DFL1, DOR, EBF2, EFE, EIN2, ERF-1, ERF1, ERF13, ERF9, ESE3, ETR2, ETT, FER, GA3OX1, GP ALPHA 1, GT72B1, HAI2, HB-7, HK3, HK5, IAA13, IAA29,

					IAA9, IP5PII, JAZ1, JAZ10, JAZ6, LAX2, MAKR6, MGL, NAC017, NRT2.7, PAD4, PAP1, PIF3, PIN4, PRE5, RAN1, RAP2.7, RAV1, RHM1, Rap2.6L, SHY2, TDR1, TIFY10B, TPL, TRX1, WRKY6, ZFP7]
GO:0071310	cellular response to organic substance	0.000000	15	70	[ABF2, ABF4, ARF19, AT1G62660, AT1G69160, AT2G17500, AT2G30020, AT3G13980, AT4G13620, AT5G14920, AT5G52020, AT5G61890, ATAF1, ATAUX2-11, BSK1, BSK5, BSL1, BZR1, C2, CBL9, CPK5, CRF10, CRF4, CTR1, D6PKL2, DEAR2, DFL1, DOR, EBF2, EFE, EIN2, ERF-1, ERF1, ERF13, ERF9, ESE3, ETR2, ETT, FER, GA3OX1, GP ALPHA 1, HAI2, HB-7, HK3, HK5, IAA13, IAA29, IAA9, IP5PII, JAZ1, JAZ10, JAZ6, LAX2, MAKR6, PAD4, PAP1, PIF3, PIN4, PRE5, RAN1, RAP2.7, RAV1, RHM1, Rap2.6L, SHY2, TDR1, TIFY10B, TPL, WRKY6, ZFP7]
GO:0010035	response to inorganic substance	0.000000	15	71	[AAE3, AATP1, ABCG40, ABF2, ABF4, ACO3, ACS6, AGT2, ALDH7B4, AT1G60420, AT2G17630, AT2G44060, AT3G17800, AT3G60750, AT5G27470, ATCS, CAD1, CAMBP25, CBL9, CCD1, CIPK23, CLC-D, CRK2, CTR1, DOR, EGY3, EIN2, ERD10, ERD15, ETT, FER4, GDH1, HB-7, HIPP22, HK3, HK5, HSP70, KT1, KTI1, LACS7, MEKK1, MGL, MYB15, MYB73, NAC017, NCED5, NRAMP3, NRT1.5, NRT2.7, OPR1, OPR3, OXS3, PAL1, PAP1, RD19, RD2, RD26, Rap2.6L, SIP3, SLAH3, STOP1, STZ, SUS3, THFS, TT10, WR3, WRKY33, XDH1, ZIFL1, cICDH, mtLPD1]
GO:0007165	signal transduction	0.000000	13	94	[ABF2, ABF4, APK2B, ARF19, AT1G62660, AT1G69160, AT2G17500,

					AT2G30020, AT2G34930, AT3G13980, AT3G23750, AT3G47570, AT4G13620, AT5G14920, AT5G52020, AT5G61890, ATAF1, ATAUX2-11, BSK1, BSK5, BSL1, BZR1, C2, CBL9, CIPK23, CIPK5, CPK5, CRF10, CRF4, CT-BMY, CTR1, D6PKL2, DEAR2, DFL1, DOR, EBF2, EIN2, ERF-1, ERF1, ERF13, ERF9, ESE3, ETR2, ETT, FER, GA3OX1, GP ALPHA 1, HAI2, HB-2, HB-7, HK3, HK5, HT1, IAA13, IAA29, IAA9, IP5PII, ITN1, JAZ1, JAZ10, JAZ6, LAX2, LRR XI-23, MAKR6, MEKK1, NPH3, PAD4, PAP1, PGIP1, PIF3, PIN4, PKS2, PLDP1, PRE5, RAN1, RAP2.7, RAV1, RAX2, RBOHD, RHM1, RLP33, RPT2, Rap2.6L, S6K2, SHY2, SIP3, STY17, TDR1, TIFY10B, TPL, UCNL, WAK2, WRKY6, ZFP7]
GO:0007165	signal transduction	0.000000	13	94	[ABF2, ABF4, APK2B, ARF19, AT1G62660, AT1G69160, AT2G17500, AT2G30020, AT2G34930, AT3G13980, AT3G23750, AT3G47570, AT4G13620, AT5G14920, AT5G52020, AT5G61890, ATAF1, ATAUX2-11, BSK1, BSK5, BSL1, BZR1, C2, CBL9, CIPK23, CIPK5, CPK5, CRF10, CRF4, CT-BMY, CTR1, D6PKL2, DEAR2, DFL1, DOR, EBF2, EIN2, ERF-1, ERF1, ERF13, ERF9, ESE3, ETR2, ETT, FER, GA3OX1, GP ALPHA 1, HAI2, HB-2, HB-7, HK3, HK5, HT1, IAA13, IAA29, IAA9, IP5PII, ITN1, JAZ1, JAZ10, JAZ6, LAX2, LRR XI-23, MAKR6, MEKK1, NPH3, PAD4, PAP1, PGIP1, PIF3, PIN4, PKS2, PLDP1, PRE5, RAN1, RAP2.7, RAV1, RAX2, RBOHD, RHM1, RLP33, RPT2, Rap2.6L, S6K2, SHY2, SIP3,

					STY17, TDR1, TIFY10B, TPL, UCNL, WAK2, WRKY6, ZFP7]
GO:0000160	phosphorelay signal transduction system	0.000000	27	25	[AT4G13620, AT5G52020, AT5G61890, CRF10, CRF4, CTR1, DEAR2, EBF2, EIN2, ERF-1, ERF1, ERF13, ERF9, ESE3, ETR2, FER, HK3, HK5, PAD4, RAN1, RAP2.7, RAV1, Rap2.6L, TDR1, WRKY6]
GO:0035556	intracellular signal transduction	0.000158	16	36	[AT4G13620, AT5G52020, AT5G61890, CIPK23, CIPK5, CPK5, CRF10, CRF4, CTR1, D6PKL2, DEAR2, EBF2, EIN2, ERF-1, ERF1, ERF13, ERF9, ESE3, ETR2, FER, HK3, HK5, HT1, MEKK1, PAD4, PLDP1, RAN1, RAP2.7, RAV1, Rap2.6L, S6K2, SIP3, STY17, TDR1, UCNL, WRKY6]
GO:0009651	response to salt stress	0.000203	15	40	[AATP1, ABF2, ABF4, ACO3, ALDH7B4, AOC3, AT1G03220, AT1G53210, AT3G23600, AT3G60750, AT4G37530, AT5G14920, C2, CAMBP25, CP1, CPL4, ChiC, EIN2, GDH1, GT72B1, HB-1, HK3, ITN1, KT1, KTI1, LACS7, MEKK1, MYB15, MYB78, NAC083, PAP1, RD19, Rap2.6L, S6K2, SIP3, SLAH3, STZ, TPPD, WRKY33, cICDH]
GO:0009733	response to auxin	0.000267	18	29	[ACS6, ARF19, AT1G69160, AT1G75580, AT2G17500, AT2G21210, AT2G37030, AT3G12955, AT3G13980, AT4G38840, ATAUX2-11, D6PKL2, DFL1, EIN2, ETT, GH3.1, HB-2, IAA13, IAA29, IAA9, LAX2, MYB15, PAP1, PIN4, RHM1, SHY2, TPL, TT4, WES1]
GO:0006970	response to osmotic stress	0.000299	14	43	[AATP1, ABF2, ABF4, ACO3, ALDH7B4, AOC3, AT1G03220, AT1G53210, AT3G23600, AT3G60750, AT4G37530, AT5G14920, BZIP53, C2, CAMBP25, CP1, CPL4, ChiC, EIN2, GDH1, GT72B1, HB-1, HK3, ITN1, KCS2, KT1, KTI1, LACS7, MEKK1, MYB15, MYB78, NAC083, PAP1, RBOHD,

					RD19, Rap2.6L, S6K2, SIP3, SLAH3, STZ, TPPD, WRKY33, cICDH]
GO:0009414	response to water deprivation	0.000343	17	30	[AATP1, ABF2, ABF4, ALDH7B4, AT2G44060, CAMBP25, CBL9, CCD1, CIPK23, DOR, ERD10, ERD15, HB-7, HK3, KT1, MGL, MYB15, NCED5, PAL1, RD19, RD2, RD26, Rap2.6L, SIP3, SLAH3, STZ, SUS3, WRKY33, XDH1, ZIFL1]
GO:0051707	response to other organism	0.001016	12	59	[AAE3, ABCG40, ADR1-L1, AOC3, APS2, AT1G59740, AT1G62660, AT2G26440, AT2G30020, AT2G34930, AT3G51450, AT5G38030, AT5G52020, AT5G52450, AT5G61890, ATAF2, ATBFRUCT1, BLH1, C2, CAD1, CAF1b, CES101, CRK8, CYP81D5, DHS1, DMR6, EFE, EIN2, ELI3-2, ERD15, FER, GDU3, HK3, HK5, HSP70, ILL6, JAZ1, JAZ10, KTI1, LAX2, NRAMP3, OPR3, PAD4, PAP1, PBF1, RBOHD, RD19, RING1, SDR3, SYTA, TLP1, TPS10, TPS21, UGT73B3, UGT73B4, UGT76B1, WRKY33, YLS2, cICDH]
GO:1901362	organic cyclic compound biosynthetic process	0.001339	10	122	[ABF2, ABF4, ADT6, AHL1, APS2, ARF19, AT1G68810, AT2G38660, AT4G13620, AT4G25800, AT5G27470, AT5G28300, AT5G49700, AT5G51910, AT5G52020, AT5G57580, AT5G61890, AT5G66120, ATAF1, ATAF2, ATAUX2-11, BBX21, BEE1, BGLU45, BLH1, BLH7, BZIP53, BZR1, C4H, CAD1, CAF1b, CIA2, CNX2, CPL4, CRF10, CRF4, CYP710A1, CYP722A1, CYP98A3, DEAR2, DHS1, ELI3-2, EMB1144, ERF-1, ERF1, ERF13, ERF9, ESE3, ESK1, ETT, FBH4, GALK, GCH, GIF2, GP ALPHA 1, GT72B1, HB-1, HB-2, HB-7, HEC1, HSF4, HSP70, IAA13, IAA29, IAA9, IDD2, IPK2BETA, JAZ1, JAZ10,

					JAZ6, KCS2, LBD16, MBD1, MYB15, MYB73, MYB77, MYB78, NAC017, NAC028, NAC032, NAC083, NAC103, NPH3, NUDX15, OMT1, PAD4, PAL1, PAL2, PAP1, PAT1, PDS1, PIF3, PRE5, RAP2.7, RAV1, RAX2, RD26, RHM1, Rap2.6L, SCL8, SHY2, SMT1, SPT, STOP1, STZ, TAT7, TDR1, THFS, TIFY10B, TLP1, TLP6, TPL, TT10, TT4, UGE3, UPB1, UPS2, UXS4, WES1, WRKY33, WRKY6, ZFP7]
GO:0009753	response to jasmonic acid	0.001395	19	22	[ABCG40, ACS6, AT3G51450, ATAF2, ChiC, EIN2, ERF1, ILL6, JAZ1, JAZ10, JAZ6, MYB15, MYB73, PAD4, PAP1, RAX2, Rap2.6L, TIFY10B, TPL, TPS10, TT4, YLS2]
GO:0044282	small molecule catabolic process	0.002199	21	19	[AAE3, ACX2, AGT2, AT4G33150, ATGA2OX1, CSY2, HGO, IBR3, IP5PII, IVD, MGL, MIOX1, MIOX2, PAL1, PAL2, PDS1, PMDH1, TAT7, THA1]
GO:0019438	aromatic compound biosynthetic process	0.002329	10	115	[ABF2, ABF4, ADT6, AHL1, APS2, ARF19, AT1G68810, AT2G38660, AT4G13620, AT4G25800, AT5G27470, AT5G28300, AT5G49700, AT5G51910, AT5G52020, AT5G57580, AT5G61890, AT5G66120, ATAF1, ATAF2, ATAUX2-11, BBX21, BEE1, BGLU45, BLH1, BLH7, BZIP53, BZR1, C4H, CAD1, CAF1b, CIA2, CPL4, CRF10, CRF4, CYP98A3, DEAR2, DHS1, ELI3-2, EMB1144, ERF-1, ERF1, ERF13, ERF9, ESE3, ESK1, ETT, FBH4, GALK, GIF2, GP ALPHA 1, GT72B1, HB-1, HB-2, HB-7, HEC1, HSF4, HSP70, IAA13, IAA29, IAA9, IDD2, IPK2BETA, JAZ1, JAZ10, JAZ6, KCS2, LBD16, MBD1, MYB15, MYB73, MYB77, MYB78, NAC017, NAC028, NAC032, NAC083, NAC103, NPH3, NUDX15, OMT1,

					PAD4, PAL1, PAL2, PAP1, PAT1, PIF3, PRE5, RAP2.7, RAV1, RAX2, RD26, RHM1, Rap2.6L, SCL8, SHY2, SPT, STOP1, STZ, TDR1, THFS, TIFY10B, TLP1, TLP6, TPL, TT10, TT4, UGE3, UPB1, UPS2, UXS4, WES1, WRKY33, WRKY6, ZFP7]
GO:0050794	regulation of cellular process	0.002579	9	166	[ABF2, ABF4, AHL1, APK2B, APS2, ARF19, ARK3, AT1G60420, AT1G62660, AT1G68810, AT1G69160, AT2G17500, AT2G30020, AT2G34930, AT2G44130, AT3G13980, AT3G23750, AT3G47570, AT4G13620, AT4G25800, AT5G10010, AT5G14920, AT5G28300, AT5G49700, AT5G51910, AT5G52020, AT5G57580, AT5G61890, ATAF1, ATAF2, ATAUX2-11, BBX21, BEE1, BLH1, BLH7, BSK1, BSK5, BSL1, BZIP53, BZR1, C2, CAD1, CAF1b, CAMBP25, CBL9, CIA2, CIPK23, CIPK5, CPK5, CPL4, CRF10, CRF4, CT-BMY, CTR1, D6PKL2, DEAR2, DEG7, DFL1, DOR, EBF2, EIN2, ELF5A-1, ERF-1, ERF1, ERF13, ERF9, ERMO2, ESE3, ESK1, ETR2, ETT, FBH4, FER, GA3OX1, GALK, GB2, GIF2, GP ALPHA 1, GRXC1, HAI2, HB-1, HB-2, HB-7, HEC1, HK3, HK5, HSF4, HSP70, HT1, IAA13, IAA29, IAA9, IDD2, IP5PII, IPK2BETA, ITN1, J20, JAZ1, JAZ10, JAZ6, KING1, KT1, LAX2, LBD16, LRR XI-23, MAKR6, MBD1, MC1, MEKK1, MYB15, MYB73, MYB77, MYB78, NAC017, NAC028, NAC032, NAC083, NAC103, NPH3, NRAMP3, PAD4, PAP1, PAPS1, PAT1, PGIP1, PIF3, PIN4, PKS2, PLDP1, PMDH1, PRE5, RABB1C, RAN1, RAP2.7, RAV1, RAX2, RBOHD, RD26, RHM1, RING1, RLP33, RPT2, Rap2.6L, S6K2, SCL8,

					SHY2, SIP3, SPT, STOP1, STY17, STZ, TDR1, TIFY10B, TLP1, TLP6, TPL, TRM5, TRX1, UCNL, UPB1, WAK2, WES1, WRKY33, WRKY6, ZFP7, ZIFL1]
GO:0050794	regulation of cellular process	0.002579	9	166	[ABF2, ABF4, AHL1, APK2B, APS2, ARF19, ARK3, AT1G60420, AT1G62660, AT1G68810, AT1G69160, AT2G17500, AT2G30020, AT2G34930, AT2G44130, AT3G13980, AT3G23750, AT3G47570, AT4G13620, AT4G25800, AT5G10010, AT5G14920, AT5G28300, AT5G49700, AT5G51910, AT5G52020, AT5G57580, AT5G61890, ATAF1, ATAF2, ATAUX2-11, BBX21, BEE1, BLH1, BLH7, BSK1, BSK5, BSL1, BZIP53, BZR1, C2, CAD1, CAF1b, CAMBP25, CBL9, CIA2, CIPK23, CIPK5, CPK5, CPL4, CRF10, CRF4, CT-BMY, CTR1, D6PKL2, DEAR2, DEG7, DFL1, DOR, EBF2, EIN2, ELF5A-1, ERF-1, ERF1, ERF13, ERF9, ERMO2, ESE3, ESK1, ETR2, ETT, FBH4, FER, GA3OX1, GALK, GB2, GIF2, GP ALPHA 1, GRXC1, HAI2, HB-1, HB-2, HB-7, HEC1, HK3, HK5, HSF4, HSP70, HT1, IAA13, IAA29, IAA9, IDD2, IP5PII, IPK2BETA, ITN1, J20, JAZ1, JAZ10, JAZ6, KING1, KT1, LAX2, LBD16, LRR XI-23, MAKR6, MBD1, MC1, MEKK1, MYB15, MYB73, MYB77, MYB78, NAC017, NAC028, NAC032, NAC083, NAC103, NPH3, NRAMP3, PAD4, PAP1, PAPS1, PAT1, PGIP1, PIF3, PIN4, PKS2, PLDP1, PMDH1, PRE5, RABB1C, RAN1, RAP2.7, RAV1, RAX2, RBOHD, RD26, RHM1, RING1, RLP33, RPT2, Rap2.6L, S6K2, SCL8, SHY2, SIP3, SPT, STOP1, STY17, STZ, TDR1, TIFY10B, TLP1, TLP6, TPL,

					TRM5, TRX1, UCNL, UPB1, WAK2, WES1, WRKY33, WRKY6, ZFP7, ZIFL1]
GO:0046395	carboxylic acid catabolic process	0.002922	23	16	[AAE3, ACX2, AGT2, AT4G33150, ATGA2OX1, CSY2, HGO, IBR3, IVD, MGL, PAL1, PAL2, PDS1, PMDH1, TAT7, THA1]
GO:0071229	cellular response to acid chemical	0.004544	14	34	[ABF2, ABF4, AT1G62660, AT2G30020, AT5G14920, ATAF1, C2, CBL9, CPK5, DOR, EFE, EIN2, ERF1, FER, GA3OX1, GP ALPHA 1, HAI2, HB-7, HK3, HK5, IP5PII, JAZ1, JAZ10, JAZ6, MAKR6, MGL, NRT2.7, PAD4, PAP1, PIF3, PRE5, TIFY10B, TPL, ZFP7]
GO:0006355	regulation of transcription, DNA-templated	0.004629	10	87	[ABF2, ABF4, AHL1, ARF19, AT1G68810, AT4G13620, AT4G25800, AT5G28300, AT5G49700, AT5G51910, AT5G52020, AT5G57580, AT5G61890, ATAF1, ATAF2, ATAUX2-11, BBX21, BEE1, BLH1, BLH7, BZIP53, BZR1, CAF1b, CIA2, CPL4, CRF10, CRF4, DEAR2, ERF-1, ERF1, ERF13, ERF9, ESE3, ESK1, ETT, FBH4, GALK, GIF2, HB-1, HB-2, HB-7, HEC1, HSF4, HSP70, IAA13, IAA29, IAA9, IDD2, IPK2BETA, JAZ1, JAZ10, JAZ6, LBD16, MBD1, MYB15, MYB73, MYB77, MYB78, NAC017, NAC028, NAC032, NAC083, NAC103, NPH3, PAP1, PAT1, PIF3, PRE5, RAP2.7, RAV1, RAX2, RD26, Rap2.6L, SCL8, SHY2, SPT, STOP1, STZ, TDR1, TIFY10B, TLP1, TLP6, TPL, UPB1, WRKY33, WRKY6, ZFP7]
GO:0033993	response to lipid	0.005058	13	47	[AATP1, ABF2, ABF4, ACO3, ALDH7B4, AT1G62660, AT2G25070, AT2G30020, AT5G14920, ATAF1, BLH1, BRH1, BSK1, BSK5, BSL1, BZR1, C2, CBL9, CP1, CPK5, ChiC, DOR, EFE, EIN2, ERD10, EXO, FER, GA3OX1, GP ALPHA 1, HAI2, HB-7, HK3,

					HK5, IP5PII, MAKR6, MYB73, MYB78, NAC083, PIF3, PRE5, RAV1, RAX2, RD26, Rap2.6L, SK32, STZ, ZFP7]
GO:0043436	oxoacid metabolic process	0.005297	11	64	[AAE3, ABF4, ACC1, ACO3, ACX2, ADT6, AGT2, AOC3, APS2, AT1G68570, AT2G17630, AT2G38660, AT4G33150, AT5G08570, AT5G27470, AT5G52020, AT5G66120, ATCS, ATGA2OX1, CAD1, CAMBP25, CSY2, CTR1, CYP81D5, D2HGDH, DHS1, EMB1144, ERF9, FAD2, GA3OX1, GDH1, GP ALPHA 1, HGO, IBR3, ILL6, IVD, J20, KCS11, KCS2, LACS7, MEE32, MGL, MIOX1, MIOX2, MYB73, NCED5, NRT2.7, OPR1, OPR3, PAD4, PAL1, PAL2, PANC, PDS1, PMDH1, Rap2.6L, SDRB, SERAT2;1, TAT7, THA1, THFS, WR3, ZFP7, cICDH]
GO:0009734	auxin-activated signaling pathway	0.008426	20	17	[ARF19, AT1G69160, AT2G17500, AT3G13980, ATAUX2-11, D6PKL2, DFL1, EIN2, ETT, IAA13, IAA29, IAA9, LAX2, PAP1, PIN4, RHM1, SHY2]
GO:0006979	response to oxidative stress	0.011544	15	29	[ABCG40, ACO3, ACS6, AT3G10020, AT3G17800, AT4G37530, AT5G16990, CRK2, DIN10, EGY3, EIN2, FER4, HK5, HSP70, KTI1, LACS7, LRR XI-23, NAC017, OPR3, OXS3, PAL1, PAL2, PAP1, SIP2, STZ, TPPD, TRX1, TT4, XDH1]
GO:1901701	cellular response to oxygen-containing compound	0.011975	13	40	[ABF2, ABF4, AT1G62660, AT2G30020, AT5G14920, ATAF1, BSK1, BSK5, BSL1, BZR1, C2, CBL9, CPK5, CTR1, DOR, EFE, EIN2, ERF1, FER, GA3OX1, GP ALPHA 1, HAI2, HB-7, HK3, HK5, IP5PII, JAZ1, JAZ10, JAZ6, MAKR6, MGL, NAC017, NRT2.7, PAD4, PAP1, PIF3, PRE5, TIFY10B, TPL, ZFP7]

GO:1901606	alpha-amino acid catabolic process	0.013156	29	10	[AGT2, AT4G33150, HGO, IVD, MGL, PAL1, PAL2, PDS1, TAT7, THA1]
GO:0009072	aromatic amino acid family metabolic process	0.014192	27	11	[ADT6, APS2, AT5G66120, DHS1, EMB1144, GP ALPHA 1, HGO, PAL1, PAL2, PDS1, TAT7]
GO:0006558	L-phenylalanine metabolic process	0.014883	50	6	[ADT6, GP ALPHA 1, HGO, PAL1, PAL2, PDS1]
GO:0071396	cellular response to lipid	0.016522	15	28	[ABF2, ABF4, AT1G62660, AT2G30020, AT5G14920, ATAF1, BSK1, BSK5, BSL1, BZR1, C2, CBL9, CPK5, DOR, EFE, EIN2, FER, GA3OX1, GP ALPHA 1, HAI2, HB-7, HK3, HK5, IP5PII, MAKR6, PIF3, PRE5, ZFP7]
GO:0009889	regulation of biosynthetic process	0.018768	10	95	[ABF2, ABF4, AHL1, APS2, ARF19, AT1G68810, AT4G13620, AT4G25800, AT5G28300, AT5G49700, AT5G51910, AT5G52020, AT5G57580, AT5G61890, ATAF1, ATAF2, ATAUX2-11, BBX21, BEE1, BLH1, BLH7, BZIP53, BZR1, CAF1b, CIA2, CPL4, CRF10, CRF4, DEAR2, ELF5A-1, ERF-1, ERF1, ERF13, ERF9, ERMO2, ESE3, ESK1, ETT, FBH4, GALK, GIF2, HB-1, HB-2, HB-7, HEC1, HSF4, HSP70, IAA13, IAA29, IAA9, IDD2, IPK2BETA, J20, JAZ1, JAZ10, JAZ6, LBD16, MBD1, MYB15, MYB73, MYB77, MYB78, NAC017, NAC028, NAC032, NAC083, NAC103, NPH3, PAD4, PAP1, PAT1, PIF3, PRE5, RAP2.7, RAV1, RAX2, RD26, Rap2.6L, S6K2, SCL8, SHY2, SPT, STOP1, STZ, TDR1, TIFY10B, TLP1, TLP6, TPL, TT4, UPB1, WES1, WRKY33, WRKY6, ZFP7]
GO:2000112	regulation of cellular macromolecule biosynthetic process	0.022340	10	90	[ABF2, ABF4, AHL1, ARF19, AT1G68810, AT4G13620, AT4G25800, AT5G28300, AT5G49700, AT5G51910, AT5G52020, AT5G57580, AT5G61890, ATAF1, ATAF2, ATAUX2-11, BBX21, BEE1, BLH1,

					BLH7, BZIP53, BZR1, CAF1b, CIA2, CPL4, CRF10, CRF4, DEAR2, ELF5A-1, ERF-1, ERF1, ERF13, ERF9, ERMO2, ESE3, ESK1, ETT, FBH4, GALK, GIF2, HB-1, HB-2, HB-7, HEC1, HSF4, HSP70, IAA13, IAA29, IAA9, IDD2, IPK2BETA, JAZ1, JAZ10, JAZ6, LBD16, MBD1, MYB15, MYB73, MYB77, MYB78, NAC017, NAC028, NAC032, NAC083, NAC103, NPH3, PAP1, PAT1, PIF3, PRE5, RAP2.7, RAV1, RAX2, RD26, Rap2.6L, S6K2, SCL8, SHY2, SPT, STOP1, STZ, TDR1, TIFY10B, TLP1, TLP6, TPL, UPB1, WRKY33, WRKY6, ZFP7]
GO:0019219	regulation of nucleobase-containing compound metabolic process	0.022503	10	88	[ABF2, ABF4, AHL1, ARF19, AT1G68810, AT4G13620, AT4G25800, AT5G28300, AT5G49700, AT5G51910, AT5G52020, AT5G57580, AT5G61890, ATAF1, ATAF2, ATAUX2-11, BBX21, BEE1, BLH1, BLH7, BZIP53, BZR1, CAF1b, CIA2, CPL4, CRF10, CRF4, DEAR2, ERF-1, ERF1, ERF13, ERF9, ERMO2, ESE3, ESK1, ETT, FBH4, GALK, GIF2, HB-1, HB-2, HB-7, HEC1, HSF4, HSP70, IAA13, IAA29, IAA9, IDD2, IPK2BETA, JAZ1, JAZ10, JAZ6, LBD16, MBD1, MYB15, MYB73, MYB77, MYB78, NAC017, NAC028, NAC032, NAC083, NAC103, NPH3, PAP1, PAT1, PIF3, PRE5, RAP2.7, RAV1, RAX2, RD26, Rap2.6L, SCL8, SHY2, SPT, STOP1, STZ, TDR1, TIFY10B, TLP1, TLP6, TPL, UPB1, WRKY33, WRKY6, ZFP7]
GO:0032774	RNA biosynthetic process	0.026354	10	88	[ABF2, ABF4, AHL1, ARF19, AT1G68810, AT4G13620, AT4G25800, AT5G27470, AT5G28300, AT5G49700, AT5G51910, AT5G52020, AT5G57580, AT5G61890, ATAF1,

					ATAF2, ATAUX2-11, BBX21, BEE1, BLH1, BLH7, BZIP53, BZR1, CAF1b, CIA2, CPL4, CRF10, CRF4, DEAR2, ERF-1, ERF1, ERF13, ERF9, ESE3, ESK1, ETT, FBH4, GALK, GIF2, HB-1, HB-2, HB-7, HEC1, HSF4, HSP70, IAA13, IAA29, IAA9, IDD2, IPK2BETA, JAZ1, JAZ10, JAZ6, LBD16, MBD1, MYB15, MYB73, MYB77, MYB78, NAC017, NAC028, NAC032, NAC083, NAC103, NPH3, PAP1, PAT1, PIF3, PRE5, RAP2.7, RAV1, RAX2, RD26, Rap2.6L, SCL8, SHY2, SPT, STOP1, STZ, TDR1, TIFY10B, TLP1, TLP6, TPL, UPB1, WRKY33, WRKY6, ZFP7]
GO:0009968	negative regulation of signal transduction	0.027909	25	11	[ATAF1, CTR1, DOR, EBF2, ETR2, FER, GP ALPHA 1, HAI2, HK5, PAD4, ZFP7]
GO:0010200	response to chitin	0.031457	23	12	[AT2G21210, BRH1, ERF-1, ERF13, MYB15, MYB73, MYB77, RING1, STZ, TDR1, WRKY33, WRKY6]
GO:0009074	aromatic amino acid family catabolic process	0.033551	56	5	[HGO, PAL1, PAL2, PDS1, TAT7]
GO:0006952	defense response	0.035865	11	59	[AAE3, ABCG40, ACS6, ADR1-L1, APK2B, AT2G27500, AT2G30020, AT2G34930, AT3G14470, AT5G51910, AT5G52020, AT5G61890, ATBFRUCT1, C2, CAD1, CAF1b, CES101, CRK8, CYP81D5, CYP82G1, DMR6, EFE, EIN2, ERF-1, ERF1, ERF13, ERF9, FER, HK3, HK5, ILL6, JAZ1, JAZ10, JAZ6, KTI1, MC1, MEKK1, NRAMP3, PAD4, PAL1, PAL2, PAP1, PAPS1, PAT1, PBF1, PGIP1, RBOHD, RD19, RING1, RLP33, RST1, SDR1, SDR3, TIFY10B, TLP1, UGT73B3, UGT76B1, WRKY33, cICDH]

GO:0031323	regulation of cellular metabolic process	0.044445	9	103	[ABF2, ABF4, AHL1, APS2, ARF19, AT1G68810, AT2G44130, AT4G13620, AT4G25800, AT5G14920, AT5G28300, AT5G49700, AT5G51910, AT5G52020, AT5G57580, AT5G61890, ATAF1, ATAF2, ATAUX2-11, BBX21, BEE1, BLH1, BLH7, BZIP53, BZR1, CAD1, CAF1b, CAMBP25, CIA2, CPL4, CRF10, CRF4, DEAR2, DEG7, ELF5A-1, ERF-1, ERF1, ERF13, ERF9, ERMO2, ESE3, ESK1, ETT, FBH4, GALK, GIF2, HB-1, HB-2, HB-7, HEC1, HK3, HSF4, HSP70, IAA13, IAA29, IAA9, IDD2, IPK2BETA, J20, JAZ1, JAZ10, JAZ6, KING1, LBD16, MBD1, MYB15, MYB73, MYB77, MYB78, NAC017, NAC028, NAC032, NAC083, NAC103, NPH3, NRAMP3, PAD4, PAP1, PAT1, PIF3, PMDH1, PRE5, RAP2.7, RAV1, RAX2, RD26, Rap2.6L, S6K2, SCL8, SHY2, SPT, STOP1, STZ, TDR1, TIFY10B, TLP1, TLP6, TPL, UPB1, WES1, WRKY33, WRKY6, ZFP7]
Downregulated at 6 HAT					
GO:0015979	photosynthesis	0.000000	31	37	[AT1G74470, ATPC1, ATPD, CA1, CAB1, CAB3, FAD5, FED A, GUN5, HCEF1, HCF243, LHB1B1, LHCA3, LHCA4, LHCB2.1, LHCB2.2, LHCB3, LHCB4.2, LHCB5, LHCB6, PETC, PRK, PSAE-2, PSAF, PSAH-1, PSAL, PSAN, PSB28, PSBO2, PSBP-1, PSBQ-2, PnsB3, RBCS1B, SBPASE, SIGA, TROL, ZKT]
GO:0019684	photosynthesis, light reaction	0.000000	38	25	[ATPC1, ATPD, CAB1, CAB3, FAD5, FED A, HCEF1, HCF243, LHB1B1, LHCA3, LHCA4, LHCB2.1, LHCB2.2, LHCB3, LHCB4.2, LHCB5, LHCB6, PETC, PSAL, PSAN, PSBO2, PSBP-1, PnsB3, TROL, ZKT]

GO:0009765	photosynthesis, light harvesting	0.000000	55	12	[CAB1, CAB3, LHB1B1, LHCA3, LHCA4, LHCB2.1, LHCB2.2, LHCB3, LHCB4.2, LHCB5, LHCB6, ZKT]
GO:0006091	generation of precursor metabolites and energy	0.000000	17	30	[ADG1, AT3G52990, ATPC1, ATPD, CAB1, CAB3, FAD5, FBA2, FED A, FdC1, HCEF1, HCF243, LHB1B1, LHCA3, LHCA4, LHCB2.1, LHCB2.2, LHCB3, LHCB4.2, LHCB5, LHCB6, PETC, PHS1, PSAL, PSAN, PSBO2, PSBP-1, PnsB3, TROL, ZKT]
GO:0018298	protein-chromophore linkage	0.000002	50	10	[CAB1, LHB1B1, LHCA3, LHCA4, LHCB2.1, LHCB2.2, LHCB3, LHCB4.2, LHCB5, LHCB6]
GO:0009416	response to light stimulus	0.000051	11	44	[ABCB1, ADG1, AT1G03010, AT1G52770, BIM1, CAB1, CAB3, CGA1, COL2, CaS, DWF1, FAD5, FED A, GATA9, HCF107, HPR, JAR1, KCS19, LHB1B1, LHCA3, LHCA4, LHCB2.1, LHCB2.2, LHCB3, LHCB4.2, LHCB5, LHCB6, MYB48, NPQ4, PETC, PHS1, PORA, PSBO2, RBCS1B, RCA, RGA1, RPL23AB, RPT2, RR5, SIGA, TK1a, ZFP1, ZKT, mtLPD1]
GO:0009735	response to cytokinin	0.003575	15	18	[ADG1, ARR9, AT1G80440, AT3G26040, ATPD, BIP2, CAD9, CGA1, HCEF1, HEME2, KASI, LHCA3, LHCB4.2, PDE334, PRK, PSAE-2, RR5, WOL]
GO:0009644	response to high light intensity	0.003618	25	10	[FAD5, LHCA3, LHCA4, LHCB2.1, LHCB2.2, LHCB3, PHS1, PSBO2, RPL23AB, ZKT]
GO:0022900	electron transport chain	0.004129	22	11	[ATPC1, ATPD, FAD5, FED A, FdC1, HCEF1, PETC, PSAN, PSBO2, PnsB3, TROL]
GO:0015995	chlorophyll biosynthetic process	0.028457	22	9	[AT1G74470, CGA1, CLA1, GSA2, GUN4, GUN5, HEMD, HEME2, PORA]
GO:0009725	response to hormone	0.029934	8	59	[ABCB1, ABCG40, ADG1, AFP1, AFP2, AIR3, AIR9, ARF8, ARR9, AT1G80440, AT3G26040, AT3G51440, AT4G30410, ATPD, AUX1, AtHB23, BIM1, BIP2, CAD9, CBF4, CGA1, COPT5, EEL,

					FBA2, FOLK, GSH2, HCEF1, HDA6, HEME2, HERK1, HHP1, HK1, JAR1, KASI, KT2/3, LHCA3, LHCB2.2, LHCB3, LHCB4.2, MYB15, PDE334, PHS1, PIP2;4, PIP2B, PORA, PRK, PSAE-2, RAP2.7, RCA, RD22, RGA1, ROP10, RR5, RVE1, SOT16, TAR2, TTL1, WOL, ZFP1]
GO:0072527	pyrimidine-containing compound metabolic process	0.031799	24	8	[CLA1, ER, KCO1, PRK, THI1, THIC, THY-1, TK1a]

Table S5 Relative expression levels of 9-cis-epoxycarotenoid dioxygenase (NCED) in horseweed leaves treated with synthetic auxin herbicides compared to water-only treatment as predicted by qRT-PCR and RNA-Seq.

Time point	Herbicide	qRT-PCR	RNA-Seq
		------------------Log2FC-----------------	
1 HAT	2,4-D	4.1	4.5
	Dicamba	3.3	4.7
	Halauxifen-methyl	2.1	2.5
6 HAT	2,4-D	5.6	3.9
	Dicamba	3.9	2.9
	Halauxifen-methyl	4.3	2.6

Table S6 Differentially expressed genes discussed in text. Relative fold change (Log2FC) values from DEseq2 analysis.

Horseweed gene	Annotation	*Arabidopsis* accession	Blast E-value	1 HAT						6 HAT					
				2,4-D		Dicamba		Halauxifen-methyl		2,4-D		Dicamba		Halauxifen-methyl	
				Log_2FC	AdjP	Log_2FC	AdjP	Log_2FC	AdjP	Log_2FC	AdjP	Log_2FC	AdjP	Log_2FC	AdjP
hw49852	IAA13	AT2G33310	9.68E-50	**1.7**	3.2E-09	**2.3**	4.2E-22	**1.8**	1.4E-09	**2.9**	5.7E-25	**2.7**	1.2E-14	**2.7**	1.9E-15
hw50873	IAA29	AT4G32280	8.56E-19	**2.5**	1.7E-14	**3.6**	1.3E-16	**2.9**	1.1E-15	**4.1**	1.6E-23	**4.1**	3.4E-17	**5.0**	3.3E-26
hw49729	IAA9	AT5G65670	4.57E-64	**0.9**	0.029	0.27	0.65	0.8	0.28	**2.3**	6.8E-27	**1.9**	5.2E-08	**2.3**	2.0E-15
hw32535	IAA13	AT2G33310	6.00E-27	**1.5**	0.001	0.78	0.29	1.1	0.31	**3.3**	1.4E-08	**3.1**	1.72E-06	**2.9**	1.7E-05
hw27084	IAA13	AT2G33310	1.59E-46	1.3	0.002	1.4	0.0035	1.4	0.005	**3.4**	1.3E-05	**3.1**	7.7E-06	**3.2**	1.5E-06
hw13742	IAA32	AT2G01200	3.34E-13	0.7	NA	NA	NA	0.5	NA	**4.0**	2.6E-07	**2.8**	4.2E-03	**3.4**	4.7E-04
hw5689	IAA3/SHY2	AT1G04240	2.09E-67	0.6	0.66	1.3	NA	0.9	0.56	**2.7**	1.7E-03	1.9	0.064	1.9	0.032
hw33543	NCED4	AT4G19170	0	-0.03	NA	0.22	NA	0.6	0.84	-0.52	NA	1.8	0.11	**2.6**	1.8E-03
hw34273	NCED5	AT1G30100	0	**4.5**	6.3E-35	**4.7**	5.5E-30	**2.5**	9.5E-07	**3.9**	3.5E-14	**2.9**	2.0E-06	**2.6**	1.2E-05
hw21726	CCD1	AT3G63520	2.21E-31	**-1.0**	0.041	0.33	0.65	0.2	0.91	**3.9**	4.7E-90	**4.3**	7.0E-52	**4.5**	5.4E-84
hw42905	ACS6	AT4G11280	4.30E-138	-0.8	0.072	0.26	0.62	-0.06	0.99	**1.1**	7.1E-03	**1.2**	3.8E-03	**1.4**	1.7E-04
hw7316	ACS10	AT1G62960	3.64E-113	0.2	0.77	0.41	0.44	0.04	0.99	**-1.8**	1.7E-17	**-1.2**	1.2E-03	**-1.8**	5.1E-05
hw15637	EFE/ACO4	AT1G05010	3.11E-117	-0.4	0.68	0.40	0.48	0.12	0.97	**-2.6**	1.1E-07	**-2.9**	1.0E-09	**-2.6**	1.4E-19
hw53227	GH3.1	AT2G14960	0	**3.5**	9.9E-13	**2.8**	4.6E-5	**2.1**	2.9E-04	**5.6**	1.7E-12	**4.8**	1.0E-08	**6.1**	1.1E-20
hw7406	WES1	AT4G27260	0	1.5	NA	**3.0**	3.2E-5	**2.7**	7.9E-08	**6.5**	5.8E-40	**6.3**	5.7E-36	**6.6**	9.2E-37
hw8261	GH3.1	AT2G14960	0	**3.3†**	0.016	1.34	NA	**4.9†**	0.046	**10.8**	4.5E-84	**10.4**	1.0E-89	**10.5**	1.9E-101
hw16576	GH3.1	AT2G14960	0	2.4	2.2E-0.4	1.26	NA	0.9	0.50	**6.0**	9.8E-21	**5.6**	2.8E-16	**3.2**	8.2E-04
hw2486	GH3.11/JAR1	AT2G46370	0	-1.5	0.015	-0.9	0.16	-0.7	0.68	**-1.4**	4.4E-13	**-1.0**	3.7E-04	**-0.8**	5.8E-03
hw21289	GH3.6/DFL1	AT5G54510	0	-0.008	1.0	-0.3	0.81	-0.2	0.98	1.4	0.15	1.0	0.50	**3.0**	7.8E-05
hw40463	GH3.5/WES1	AT4G27260	0	-1.6	0.001	0.2	0.85	0.2	0.97	**3.2**	1.6E-09	**3.2**	0.6E-08	**2.9**	7.9E-05
hw35545	GH3.10/DFL2	AT4G03400	0	-0.8	0.28	0.2	0.87	-0.3	0.94	**-2.1**	4.8E-03	0.8	NA	-0.2	0.91
hw13007	SRG1	AT1G17020	1.97E-147	-0.5	0.50	0.3	0.57	0.07	0.99	**1.2**	4.5E-07	**1.5**	3.9E-07	**2.1**	7.2E-09

Relative fold change values significant (adjusted p-value ≤ 0.05) in at least two of the three analysis methods (DEseq2, edgeR, and Cufflinks) are bolded and highlighted in blue.
NA represents genes that did not pass the filter threshold.
†Relative fold change value from edgeR analysis.
